# Longitudinal study on the effects of growth-promoting and therapeutic antibiotics on the dynamics of chicken cloacal and litter microbiomes and resistomes

**DOI:** 10.1186/s40168-021-01136-4

**Published:** 2021-08-28

**Authors:** Chhedi Lal Gupta, Shlomo E. Blum, Karuppasamy Kattusamy, Tali Daniel, Shelly Druyan, Roni Shapira, Oleg Krifucks, Yong-Guan Zhu, Xin-Yuan Zhou, Jian-Qiang Su, Eddie Cytryn

**Affiliations:** 1 Department of Soil Chemistry, Plant Nutrition and Microbiology, Institute of Soil, Water and Environmental Sciences, The Volcani Institute, Agricultural Research Organization, 7528809 Rishon LeZion, Israel; 2 Department of Bacteriology, Kimron Veterinary Institute, 50250 Beit Dagan, Israel; 3 Institute of Biochemistry, Food Science and Nutrition, The Robert H. Smith Faculty of Agriculture, Food and Environment, The Hebrew University of Jerusalem, Rehovot, Israel; 4 Institute of Animal Science, Poultry and Aquaculture, The Volcani Institute, Agricultural Research Organization, 7505101 Rishon LeZion, Israel; 5 Key Laboratory of Urban Environment and Health, Institute of Urban Environment, Chinese Academy of Sciences, Xiamen, 361021 China; 6 Research Center for Eco-environmental Sciences, Beijing, 100049 China

**Keywords:** Broiler chickens, Growth-promoting antibiotics, Shotgun metagenomics, Microbiome, Antimicrobial resistance, Antibiotic-resistant bacteria, Antibiotic resistance genes, Priority pathogens, 16S rRNA gene amplicon sequencing, HT-qPCR

## Abstract

**Background:**

Therapeutic and growth-promoting antibiotics are frequently used in broiler production. Indirect evidence indicates that these practices are linked to the proliferation of antimicrobial resistance (AMR), the spread of antibiotic-resistant bacteria from food animals to humans, and the environment, but there is a lack of comprehensive experimental data supporting this. We investigated the effects of growth promotor (bacitracin) and therapeutic (enrofloxacin) antibiotic administration on AMR in broilers for the duration of a production cycle, using a holistic approach that integrated both culture-dependent and culture-independent methods. We specifically focused on pathogen-harboring families (*Enterobacteriaceae*, *Enterococcaceae*, and *Staphylococcaceae*).

**Results:**

Antibiotic-resistant bacteria and antibiotic resistance genes were ubiquitous in chicken cloaca and litter regardless of antibiotic administration. Environment (cloaca vs. litter) and growth stage were the primary drivers of variation in the microbiomes and resistomes, with increased bacterial diversity and a general decrease in abundance of the pathogen-harboring families with age. Bacitracin-fed groups had higher levels of bacitracin resistance genes and of vancomycin-resistant *Enterococcaceae* (total *Enterococcaceae* counts were not higher). Although metagenomic analyses classified 28–76% of the *Enterococcaceae* as the commensal human pathogens *E. faecalis* and *E. faecium*, culture-based analysis suggested that approximately 98% of the vancomycin-resistant *Enterococcaceae* were avian and not human-associated, suggesting differences in the taxonomic profiles of the resistant and non-resistant strains. Enrofloxacin treatments had varying effects, but generally facilitated increased relative abundance of multidrug-resistant *Enterobacteriaceae* strains, which were primarily *E. coli*. Metagenomic approaches revealed a diverse array of *Staphylococcus* spp., but the opportunistic pathogen *S. aureus* and methicillin resistance genes were not detected in culture-based or metagenomic analyses. *Camphylobacteriaceae* were significantly more abundant in the cloacal samples, especially in enrofloxacin-treated chickens, where a metagenome-assembled *C. jejuni* genome harboring fluoroquinolone and β-lactam resistance genes was identified.

**Conclusions:**

Within a “farm-to-fork, one health” perspective, considering the evidence that bacitracin and enrofloxacin used in poultry production can select for resistance, we recommend their use be regulated. Furthermore, we suggest routine surveillance of ESBL *E. coli*, vancomycin-resistant *E. faecalis* and *E. faecium*, and fluoroquinolone-resistant *C. jejuni* strains considering their pathogenic nature and capacity to disseminate AMR to the environment.

Video Abstract

**Supplementary Information:**

The online version contains supplementary material available at 10.1186/s40168-021-01136-4.

## Background

Commercial broiler chicken facilities traditionally use large quantities of therapeutic antibiotics and antibiotic growth promotors (AGPs) to ensure flock health and increase productivity. Therapeutic antibiotics are usually administered following the outbreak of a disease, at a therapeutic dose, for a short window of time, targeting specific pathogens associated with the disease. AGPs on the other hand are feed additives given at sub-therapeutic doses for most of the growth cycle, regardless of the presence of a disease or specific pathogens, and are believed to improve weight gain [1–3]. The fluoroquinolone enrofloxacin was first licensed for the treatment of respiratory diseases in poultry in the USA in 1996, but the clinical importance of fluoroquinolones and the alarming evidence of quinolone-resistant zoonotic pathogens (e.g., *Campylobacter* spp., *Enterobacteriales*), led the USA, European Union, and other countries to ban their use in food animal production [4–7]. Nonetheless, fluoroquinolones are still broadly used in Asia, the Middle East, and South America, where a large fraction of global poultry production facilities exist. Although direct evidence linking the use of fluoroquinolones in poultry production to antimicrobial resistance (AMR) is lacking, the fact that resistance in *E. coli* from poultry in the USA is below 5%, vs. over 40% in Brazil and China where the use of fluoroquinolones in poultry is permitted [8], suggests use may be facilitating resistance. Nonetheless, banning fluoroquinolones has not completely eliminated the occurrence of resistant populations [9]. This may be explained by the fact that bacteria can remain resistant to antibiotics long after eliminating selection or due to the fact that many environmental bacteria intrinsically harbor ARGs, regardless of selective pressure [10].

It was traditionally argued that the AGPs commonly used as feed additives in food animal production (i.e., bacitracin, viriniamycin, tyosin, and avoparcin) are not a public health concern since they are generally not administered in humans. However, indirect evidence suggests that the use of AGPs can facilitate resistance to clinically relevant antibiotics through co-selection [11, 12] and cross-resistance [13]. Hegde et al. showed that chickens fed a conventional diet supplemented with AGPs had a higher abundance of specific antibiotic resistance genes (ARGs) in their gut microbiomes than chickens fed organic diets without antibiotics [14]. Such studies led to the restriction of AGP use in animal husbandry in the European Union and other countries [15–17]; however, they are still broadly used throughout the world [18]. Despite the indirect link to antimicrobial resistance, tangible (and especially quantitative) evidence on the causal association between AGP use and AMR in food animals is currently lacking. Furthermore, therapeutic and prophylactic antibiotics can modify fecal microbial communities [19] resulting in substantial shifts in ARG distribution, with both increased and decreased relative abundance of particular genes that are not directly dictated by selection [20, 21]. Therefore, concomitant investigation of microbiomes and resistomes is needed for a holistic understanding of the effects of antibiotics used in food animals.

Here, we performed a comprehensive longitudinal study on the effects of therapeutic antibiotics and AGPs on AMR in both the cloaca, as an indicator of AMR transmission via food production, and litter, as a source of AMR transmission to crops and the water cycle through fertilization. Our primary objectives were to (i) acquire a comprehensive understanding of poultry and litter microbiomes and resistomes as a function of the growth stage, (ii) assess the effect of therapeutic and prophylactic antibiotics on poultry microbiomes and resistomes, and (iii) determine the potential synergistic effects of combined use. Longitudinal sampling of individual birds was applied to provide evidence of causal effects. We hypothesized that higher rates of AMR would accumulate throughout the production cycle in groups treated with AGPs, compared to short-term antibiotic treatment or the non-treated control groups. We combined culture-dependent and culture-independent “omics” methods aiming to complement and validate the molecular data with actual resistant bacteria isolation, identification, and antibiotic resistance profiling, due to the limitations on inferring resistance inherent to metagenomics datasets. We used the fluoroquinolone enrofloxacin (EFX) and bacitracin methylene disalicylate (BMD) to simulate therapeutic and AGP use of antibiotics, respectively, because these are still widely used molecules in poultry production in many parts of the world. We focused on vancomycin-resistant enterococci (VRE), methicillin-resistant staphylococci (MRS), and extended-spectrum beta-lactamase-producing *Enterobacteriaceae* (ESBL-E) that are resistant to third-generation cephalosporins, due to the clinical importance of priority pathogens from these groups such as vancomycin-resistant *E. faecium* and *E. faecalis*, methicillin-resistant *Staphylococcus aureus*, and ESBL-producing *Klebsiella pneumonia* and *E. coli*.

## Results

The study was conducted in an experimental poultry house, simulating a full commercial broiler production cycle. Animals were divided into 12 pens, with 30 chickens in each pen. Up to day 27, half of the animals (6 pens) were fed BMD-supplemented feed at a standard AGP dose, and the other six pens received feed without antibiotics. On day 28, half of the animals (three out of six pens) in each dietary treatment group (with and without BMD) were treated for 3 days with the fluoroquinolone enrofloxacin administered through drinking water at a standard therapeutic dose. The experiment consisted therefore of four treatments: (I) no antibiotic (NAB), (II) BMD, (III) EFX, and (IV) BMD_EFX combined, each in triplicate pens with a total of 90 chickens per treatment. Cloacal swabs from 10 chickens per pen (sampling the same birds throughout the experiment) and litter samples from all of the pens were taken on day 27 (prior to EFX treatment), day 31 (immediately after EFX treatment), and on day 41 (at the end of the growth cycle). Meat (internal pectoral muscle) was sampled after slaughter. Overall, there was 7.78% cumulative mortality throughout the experiment, primarily towards the end of the growth cycle due to heat stress. Mortality was random between pens, with no significant treatment effect. No differences were found in body weight gain between the treatment groups throughout the experiment (Figure S1), and none was found in the meat processing parameters (body weight, relative heart, liver, and abdominal fat weights) between treatments after slaughter. Relative breast weight (RBW) was the only meat processing parameter with statistically significant differences: average RBW was approximately 5% higher in animals treated with enrofloxacin (both EFX and BMD_EFX treatments) compared to non-antibiotic treated animals, with the BMD-fed groups in between (Figure S2).

### Effect of growth stage and antibiotic treatment on cloacal swab and litter microbiomes

Illumina NovaSeq 6000 sequencing generated > 200 GB of data, with an average of ~ 31 M reads per composite sample. More than 10 M contigs were assembled from the cloacal and litter samples following quality control. Subsequent open reading frame (ORF) prediction resulted in about 13.6 M ORFs (~ 5.5 M non-redundant ORFs at 95% clustering threshold, Table S1). Assemblies were further binned using MetaBAT2, resulting in 368 and 222 metagenome-assembled genomes (MAGs, ranging in size from 200 kb to 10 Mb) from the cloacal swabs and litter, respectively (Table S2). These included 48 and 15 “high-quality” MAGs (according to the MIMAG criteria [22]), which were de-replicated (using the dRep workflow with a relatedness threshold of ANImf > 99), obtaining a reduced set of 26 and 12 putative genomes for swab and litter, respectively, with average estimated genome completeness of 95.10% (ranging between 90.25 and 99.20%) and mean contamination of 0.71% (ranging between 0 and 2.39%) (Table S3).

**Fig. 1:**
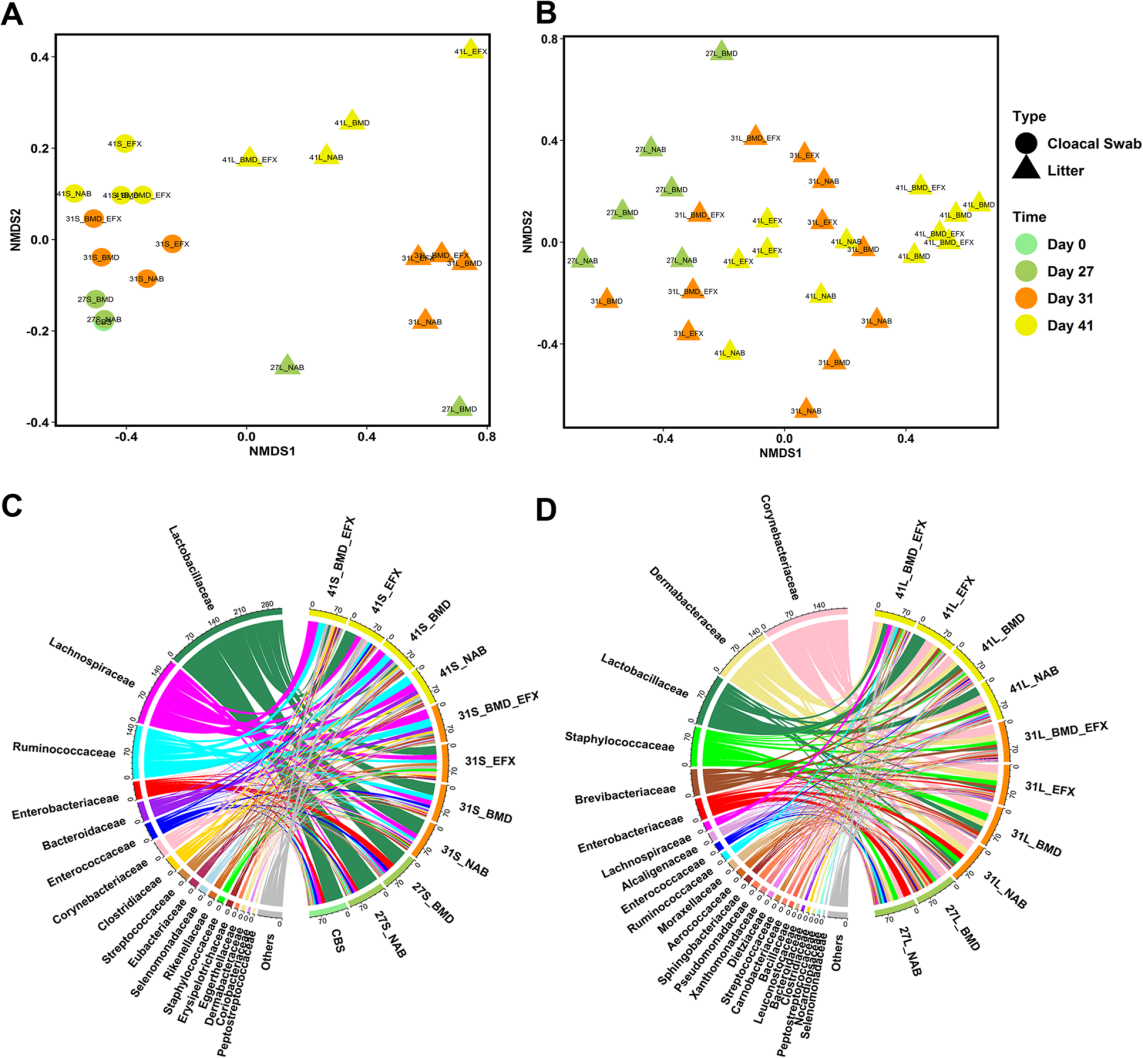
Effect of growth stage and antibiotic treatment on the bacterial community composition and diversity of poultry cloacal swab and litter. **A**, **B** Non-metric multidimensional scaling (NMDS) plots of poultry microbiomes using Bray–Curtis distance matrices, based on **A** cloacal swab and litter metagenomes (stress = 0.13), and **B** 16S rRNA gene amplicons from litter (stress = 0.10). **C**, **D** Chord diagrams depict the relative abundance of bacterial families in **C** cloacal swabs and **D** litter; unclassified taxa and bacterial families with relative abundance below 0.5% were grouped into “others.” The outer circle lists the sample names, where colors represent the sampling times, and the detected bacterial families. The connecting lines inside the circle link families to the samples, and the width of the lines is proportional to the relative abundance (%) of each family in the corresponding samples

The litter microbiome was significantly different from that of cloacal swabs (Bray–Curtis; PERMANOVA; *p* < 0.01), and a distinct temporal effect was observed in the microbial community composition of both swab and litter samples (Bray–Curtis; PERMANOVA; *p* < 0.05 and *p* < 0.01, respectively), which was much more significant than the antibiotic treatment effect (Fig. 1A). This temporal effect on the litter microbiome was also observed in 16S rRNA gene amplicon sequencing (Bray–Curtis; PERMANOVA; *p* < 0.01, Fig. 1B), where a significant increase in bacterial diversity with time was observed (*p* < 0.05; Figure S4A). Taxonomic classification of metagenomic data revealed that an average of 98.08% and 97.98% of the contigs in the cloacal swab and litter samples, respectively, was bacteria. Viruses, Eukaryota, and Archaea accounted for 1.21%, 0.07%, and 0.64% of the swabs and 1.82%, 0.18%, and 0.02% of the litter microbiomes, respectively (Figure S3A). The abundance of Archaea was higher in the cloacal swabs (0.64%) than in the litter (0.02%), whereas the opposite was true for the viruses. Viruses were predominantly bacteriophages, and almost all of the Archaea detected were methanogens (Figure S3B). The latter included an almost completely assembled MAG characterized as *Methanobrevibacter woesei* (Table S3).

Taxonomic analysis revealed that *Lactobacillaceae*, *Lachnospiraceae*, *Ruminococcaceae*, and *Enterobacteriaceae* were the most abundant bacterial families in the cloacal swabs, whereas *Corynebacteriaceae*, *Dermabacteraceae*, *Lactobacillaceae*, and *Staphylococcaceae* were dominant in the litter (Fig. 1C, D; Figure S4B). The relative abundance of *Enterobacteriaceae*, *Staphylococcaceae*, and *Enterococcaceae* decreased over time in both cloaca (Fig. 1C) and litter (*p* < 0.05; Figure S5), whereas the abundance of *Lactobacillaceae* decreased over time in the cloaca, but increased in the litter. This trend was also observed for a high-quality *Lactobacillaceae* MAG, characterized as *Ligilactobacillus agilis* (previously *Lactobacillus agilis*) (Table S3), a bacterium that is commonly found in chicken intestines and is associated with probiotic activity [23]. In the litter, the relative abundance of the Actinobacterial genera *Nocardiopsis* and *Enteractinococcus* and the proteobacterial genera *Paracoccus*, *Halomonas*, and *Pusillimonas* increased significantly with time (Figure S6A). Conversely, the relative abundance of *Escherichia/Shigella* and *Staphylococcus* decreased with time, supporting the family-level (*Enterobacteriaceae*/*Staphylococcaceae*) results described above. The impact of the antibiotic treatment was most apparent at the later stages of the poultry growth cycle (day 41; Figure S6B). The relative abundance of the Actinobacterial genera *Bogoriella* and *Georgenia* was significantly higher in the litter of BMD-supplemented chickens. BMD also facilitated increased relative abundance of certain Proteobacteria such as *Falsochrobactrum*, *Psychrobacter*, and *Pusillimonas*. EFX treatment on days 31 and 41 resulted in a significantly higher abundance of the Enterobacterial genera *Proteus* and *Providencia*, correlating with the higher relative abundance of *Enterobacteriacae* observed in swabs following EFX treatment (0.37% vs. 0.06%, day 41) in the metagenomic analysis. In contrast, BMD lowered the relative abundance of *Escherichia/Shigella* (Figure S6B).

Metagenomic-assembled ORFs were taxonomically assigned by employing the MEGAN LCA algorithm to specifically elucidate the abundance and distribution of potential priority pathogens associated with the *Staphylococcaceae*, *Enterococcaceae*, and *Enterobacteriaceae* families (Figure S7). We subsequently focused on MAGs associated with these families, inferring the taxonomy of the recovered MAGs against the Genome Taxonomy Database (GTDB) using the GTDB-Tk toolkit (see the “Methods” section). Only average nucleotide identity (ANI) scores above 95% were considered for species-level classification. Initially, 38 and 19 priority family MAGs with different levels of genome completeness (Table S2) were recovered (17 and 7 representative MAGs from swab and litter samples, respectively, following de-replicated using the dRep workflow as described above; Figure S8) and characterized to species level. The mean ANI score of identified representative MAGs was 98.3% (ranging between 95.34 and 99.96%) (Table S4). *Staphylococcus chromogenes* was highly abundant in many of the cloacal samples, whereas *S. lentus*, *S. xylosus*, and *S. arlettae* were the primary *Staphylococcaceae* species in the litter. Five *Staphylococcaceae* MAGs were identified, including nearly complete (high quality) *S. chromogenes* (98.01/0.57% genome completeness/contamination) and *S. arlettae* (97.93.22/0.55% completeness/contamination) genomes in the swab and litter, respectively. The opportunistic pathogen *S. aureus* was not detected in any of the samples analyzed. We were able to assemble 14 *Enterococcaceae* MAGs, which included *E. faecium*, *E. casseliflavus*, *E. gallinarum*, *E. durans*, *E. faecalis*, *E. hirae*, and *E. avivum.* Importantly, a nearly complete genome of *E. faecalis* (93.24/2.39% completeness/contamination) was recovered from the cloacal swabs. *Escherichia coli* was the most abundant *Enterobacteriaceae* species in both cloacal swab and litter, and four *Escherichia* spp. MAGs (most closely associated with *E. coli* and *E. flexneri*) were recovered from these samples. We also successfully recovered a *Campylobacter jejuni* MAG (73.39/0.27% completeness/contamination) from the day 31 EFX sample (Table S4). The abundance of *Camphylobacteriaceae* (*p* < 0.01) was higher in cloacal swabs compared to litter and was found increased on day 31 in EFX-treated groups relative to NAB samples (0.74% vs. 0.14%).

### Effect of growth stage and antibiotic treatment on cloacal swab and litter resistomes

**Fig. 2:**
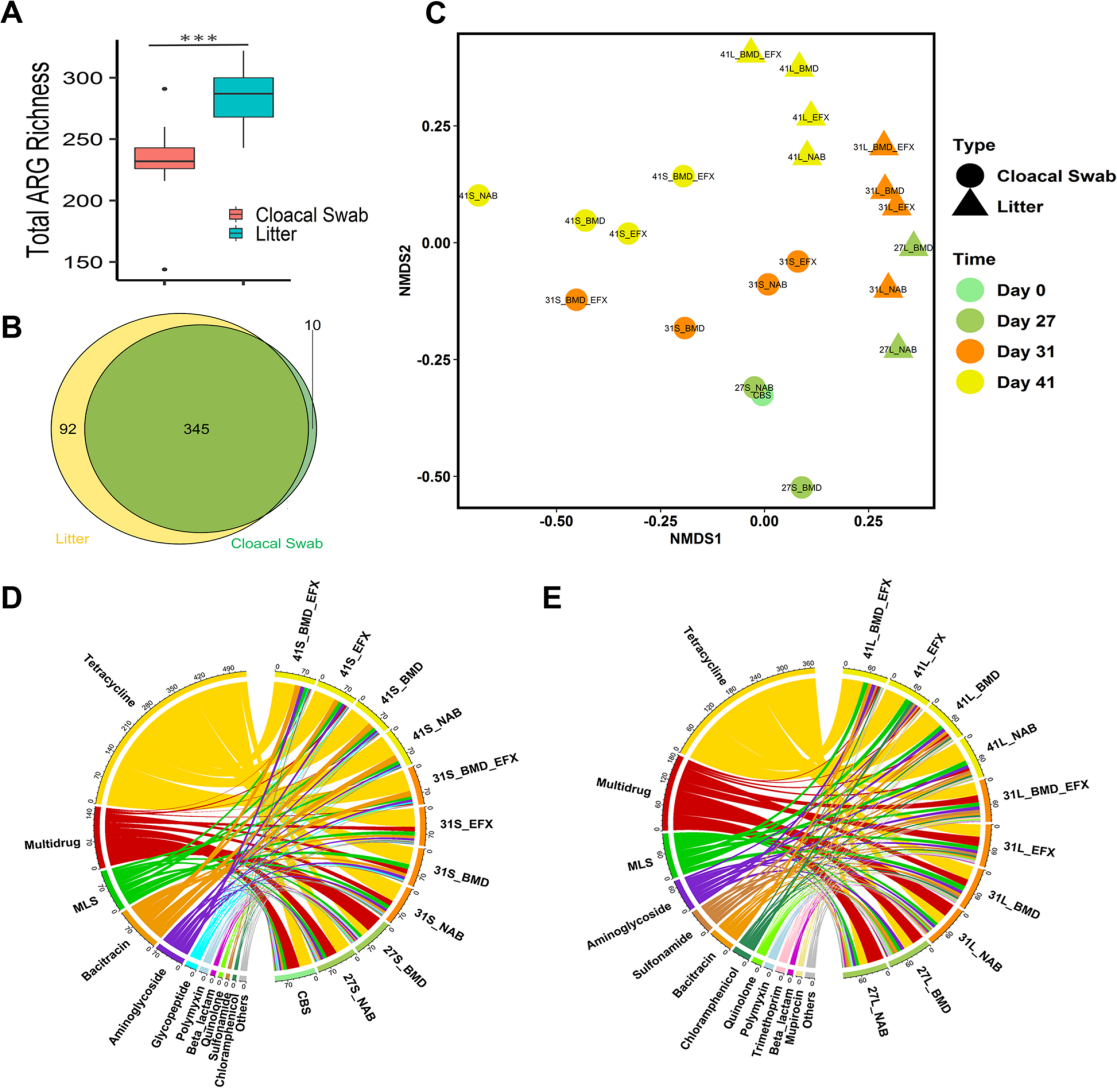
Effect of sample source (chicken cloacal swab and litter), growth stage, and antibiotic treatment on ARG composition and diversity. **A** Boxplot of the number of unique ARGs detected in cloacal swab and litter environments. **B** Venn diagram of shared and unique ARGs among cloacal swab and litter environments. **C** NMDS plot of the poultry resistome based on Bray–Curtis distance matrices (stress = 0.04). **D**, **E** Chord diagrams depict the relative abundance of ARGs in **D** cloacal swabs and **E** litter; the ARG types with relative abundance of less than 0.5% were grouped into “others.” The outmost circle lists the name of poultry samples, where colors represent sampling times, and detected ARG types. The connecting lines inside the circle links ARG types to the samples and the width of the lines is proportional to the relative abundance (%) of each ARG type in the corresponding samples. ***p < 0.001 by non-parametric *t*-test

The composition and diversity of cloacal swab and litter resistomes were extrapolated from shotgun metagenomic data by screening against the DeepARG database. In total, 355 and 437 ARG subtypes from 22 and 25 ARG classes were detected in cloacal swabs and litter, respectively (Table S5). Similar to the microbiomes, the resistome composition was strongly dictated by the environment (cloaca vs. litter, Bray–Curtis; PERMANOVA; *p* < 0.01) with ARG diversity being greater in litter than in cloacal swabs (*p* < 0.001; Fig. 2A). Almost all cloacal swab ARGs were also observed in the litter (Fig. 2B). Strong temporal shifts in cloaca and litter ARG composition were also observed (Bray–Curtis; PERMANOVA; *p* < 0.05 for swabs and *p* < 0.01 for litter, Fig. 2C). Over 65% of the ARGs detected in the swab and litter samples were associated with tetracycline, multidrug efflux pumps, and macrolide-lincosamide-streptogramin (MLS) resistance classes (Fig. 2D, E). In both swabs and litter, the relative abundance of tetracycline resistance genes increased with time, while the abundance of multidrug efflux pumps decreased (*p* < 0.05, day 41 vs. 31). An increase in the relative abundance of bacitracin and a decrease in quinolone resistance genes with time were also observed in cloacal swabs (*p* < 0.01). On day 41, the relative abundance of quinolone resistance genes was higher on EFX-treated groups relative to NAB (0.4% vs. 0.02%) in swabs, but the opposite phenomenon was observed in the litter (0.91% vs. 1.4%), suggesting that enrofloxacin concentrations selected for fluoroquinolone resistance in the chicken gut, but not in the litter. On day 31, the relative abundance of bacitracin resistance genes was higher in the BMD-fed groups relative to NAB in both swabs (9.17% vs. 6.26%) and litter (5.56% vs. 2.96%). To pinpoint high-risk ARGs, we employed ARG ranker, which categorizes ARGs into four ranks (I–IV) based on anthropogenic enrichment, mobility, and host pathogenicity [24]. Twenty-two first-ranked ARGs were identified, 50% of whom conferred resistance to macrolides (6 ARGs) and aminoglycosides (5 ARGs) (Figure S9).

We further targeted antibiotic resistance genes in the litter by applying high-throughput quantitative PCR (HT-qPCR) arrays, specifically investigating 53 clinically relevant ARGs and MGEs (mobile genetic elements), primarily detected in the shotgun metagenomic analyses. Almost all targeted ARGs and MGEs were detected by HT-qPCR array analysis, with the exception of *vanA*, *tetW*, *sul*1, and *aaC*(6’)-II (Figures S10 and S11A). On day 31, the relative abundance of the *Staphylococcus* spp.-associated multidrug efflux pump-encoding gene *mepA* was significantly higher in the BMD groups relative to the NAB control (Figure S11B). Similarly, BMD showed a significantly higher abundance of the multidrug efflux pump-encoding gene *emrD*, which was also significantly more abundant in the EFX treatments on days 31 and 41. Surprisingly, on day 41, the relative abundance of the bacitracin resistance-conferring gene *bacA*-02 was significantly lower in the BMD treatment (Figure S11B). This may stem from the fact that the bacitracin concentrations in the litter were not high enough to select for resistance or to the fact that other resistance mechanisms may have also conferred resistance to bacitracin in this setting.

### Linking ARGs to specific bacterial phyla

**Fig. 3:**
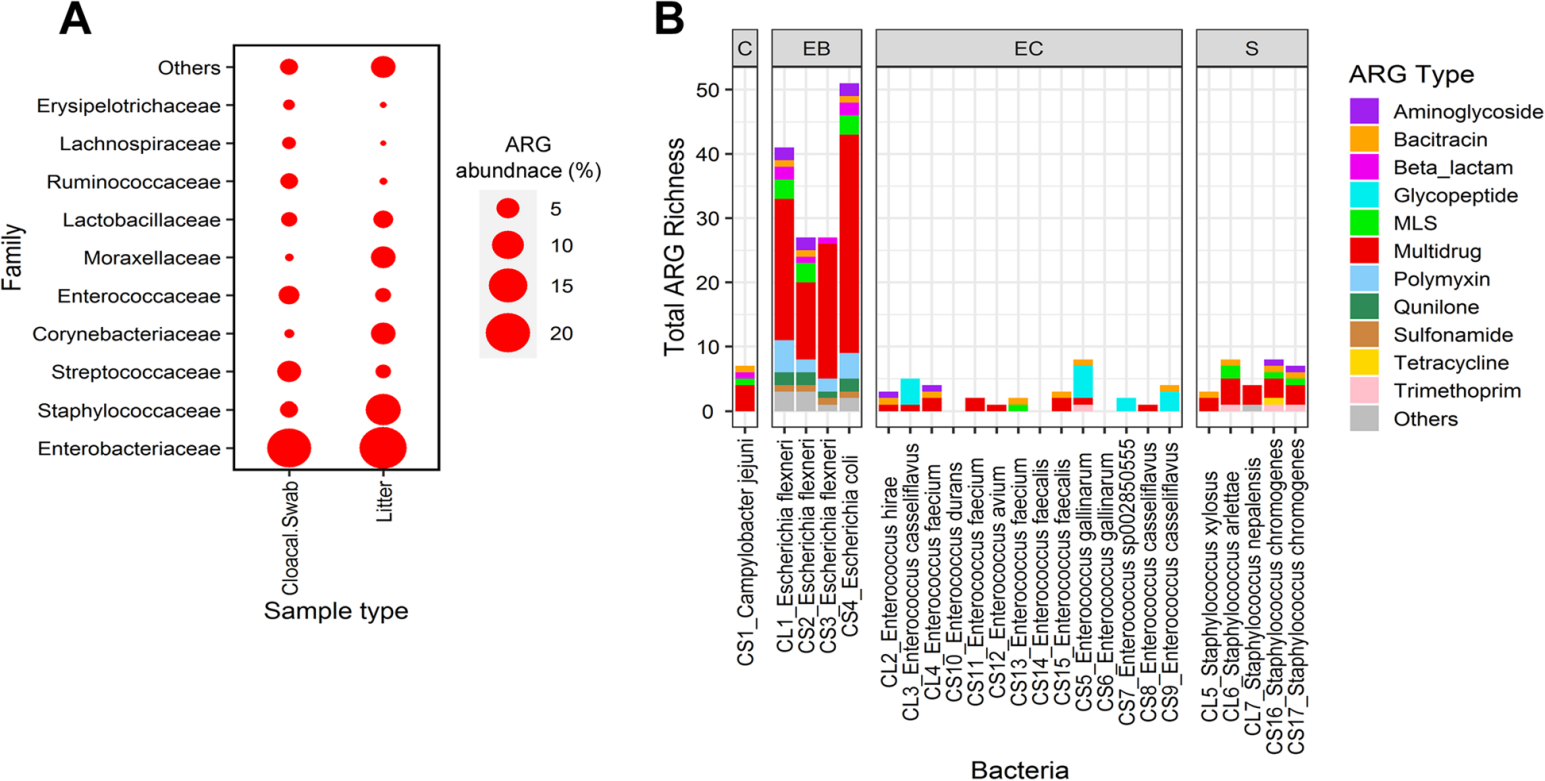
Linking ARGs to bacteria (host) in poultry swab and litter environments. **A** Predicted abundance of ARGs in selected bacterial families in chicken cloaca and litter. The first 10 families, predicted to represent 91.58% of the total ARG abundance, are listed here, while the remaining are grouped as “others.” ARG taxonomy was estimated by assigning given taxa at the family level to ARG-containing metagenomic-assembled ORFs. **B** Distribution of predicted ARGs in MAGs of priority pathogen-harboring families recovered from cloacal swabs (CS) and chicken litter (CL). C, Camphylobacteriaceae; EB, Enterobacteriaceae; EC, Enterococcaceae; S, Staphylococcaceae

We inferred the taxonomic affiliation of obtained ARGs in chicken metagenomes by assigning taxonomy at the family level to ARG-containing metagenomic-assembled ORFs (Fig. 3A). Most characterized ARGs were predominantly associated with *Enterobacteriaceae* in both cloacal swab and litter environments, undoubtedly a bias associated with the fact that genes from this family dominate ARG databases. ARGs were also significantly enriched in *Enterococcaceae* (*p* < 0.05) and *Streptococcaceae* (*p* < 0.001) in the swabs, and in *Staphylococcaceae* (*p* < 0.01) and *Moraxelleceae* (*p* < 0.*0001*) in the litter. This phenomenon was validated by the metagenome assembly, with 27–51, 3–8, and 0–8 ARGs detected in the *Enterobacteriaceae*, *Staphylococcaceae*, and *Enterococcaceae* MAGs, respectively (Fig. 3B; Table S6). Vancomycin/glycopeptide resistance-conferring genes (i.e., *vanSC*, *vanRC*, *vanTC*, *vanXYC*, and *vanC*) were detected in *E. gallinarium* and *E. casseliflavus* MAGs, but not in the human opportunistic pathogen enterococci (*E. faecium* and *E. feacalis*) genomes, which may explain the low isolation level of these species (see the “Culture-based analysis of resistant priority pathogens” section). The latter did contain multidrug efflux pumps, including *efrA* and *uppP*, conferring resistance to fluoroquinolones and bacitracin, respectively. Half of the ARGs detected in *E. coli* and *E. flexneri* genomes were multidrug efflux pumps, including *emrD* that was significantly more abundant in both antibiotic treatments (Figure S11B). *Escherichia* MAGs also contained the bacitracin resistance gene *bacA*, as well as *mrdA*, which was linked to increased carbapenem and diazabicyclooctane resistance in *E. coli* [25]. Interestingly, the assembled *Campylobacter jejuni* genome was predicted to harbor 7 ARGs, including multidrug efflux pumps conferring fluoroquinolone (*cmeA*, *cmeB*, *cmeC*, *cmeR*) and bacitracin (*uppP*) resistance and the recently identified β-lactamase *bla*_OXA-61_ [26] (Fig. 3B; Table S6).

**Fig. 4:**
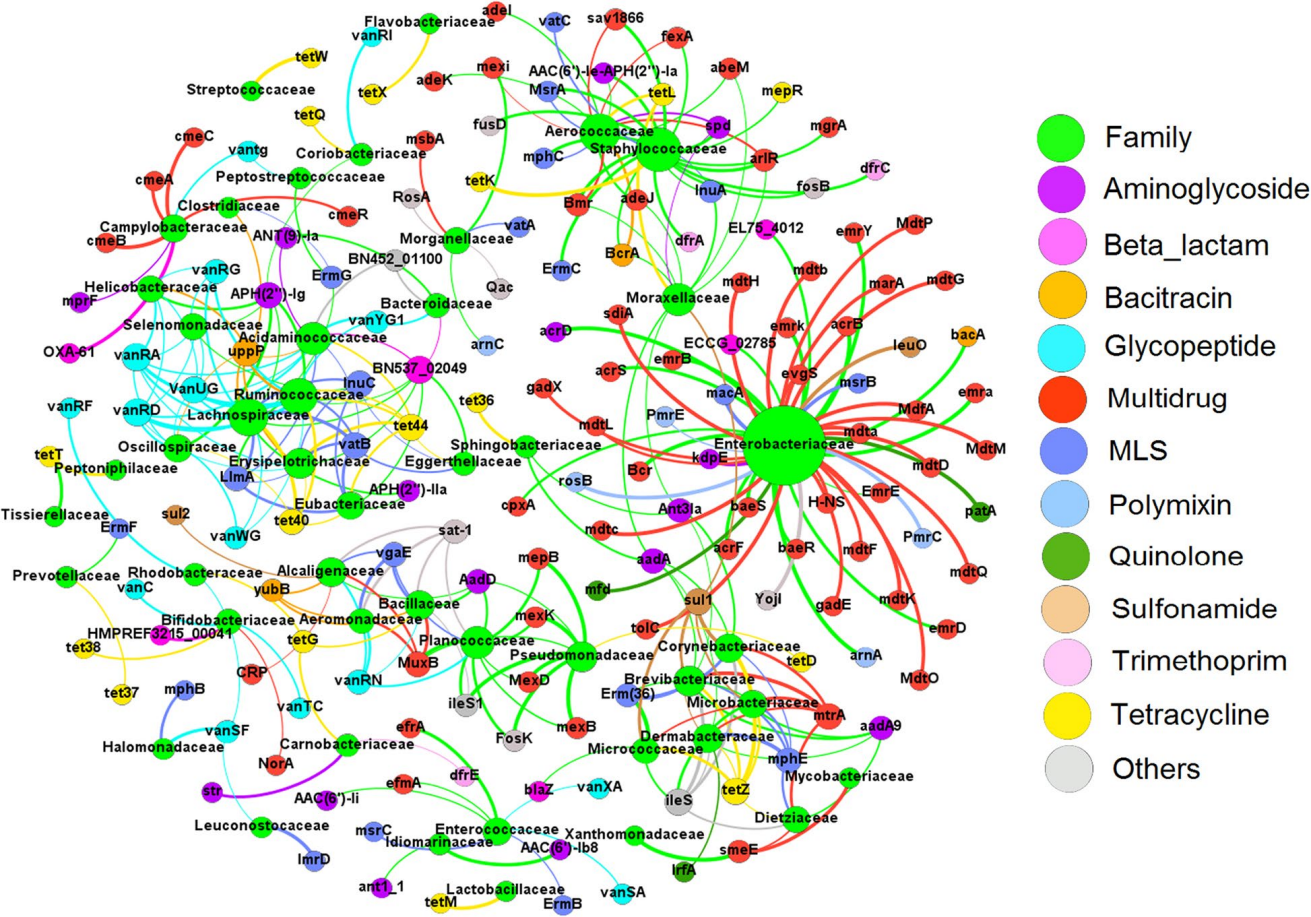
Network analysis showing correlations between ARGs and bacterial families in cloacal swab and litter. Nodes represent ARG subtypes, and/or families and edges (i.e., connections between ARG subtypes and bacterial families) indicate strong and significant (padj < 0.01) pairwise correlations (spearman rho > 0.80). The size of each node is proportional to the number of connections (i.e., degree), and the edge thickness is proportional to the Spearman correlation coefficient (rho 0.80–0.99). The network consists of 207 nodes and 320 edges with a modularity index of 0.82, indicating that the obtained network had a modular structure. The network is colored by ARG types and bacterial families. MLS, macrolide-lincosamide-streptogramin

Network analysis was applied to further investigate the underlying global associations between ARGs, MGEs, and phylogeny, focusing on the co-occurrence patterns in taxa present in at least one-third (7 out of 21) of the metagenomic samples (Fig. 4). ARGs associated with *Enterobacteriaceae* and *Enterococcaceae* were not shared with other taxa in the network, but some *Staphylococcaceae* ARGs (i.e., *tetL*) were also found in *Aeroccaceae*, and *sul1* was common to several Gram-positive bacteria. Network analysis of MGEs and ARGs revealed that the efflux pump *emrD*, which was strongly linked to *Enterobacteriaceae*, was also associated with transposases such as *tnpR* (Figure S12A), suggesting that it may be horizontally transferred. Interestingly, transposons correlated with several multidrug efflux pump-associated genes (Figure S12A), while integron-associated genes such as *intl1* and *intl2* were linked to a diverse range of bacterial families (Figure S12B). While the network provides important insights, co-occurrence network analyses are based on statistical associations (not on confirmed genetic linkage) and can result in false assumptions, therefore should be taken carefully, and if possible supported by additional evidence when attempting to link individual ARGs/MGEs to specific taxa.

### Culture-based analysis of resistant priority pathogens

**Fig. 5:**
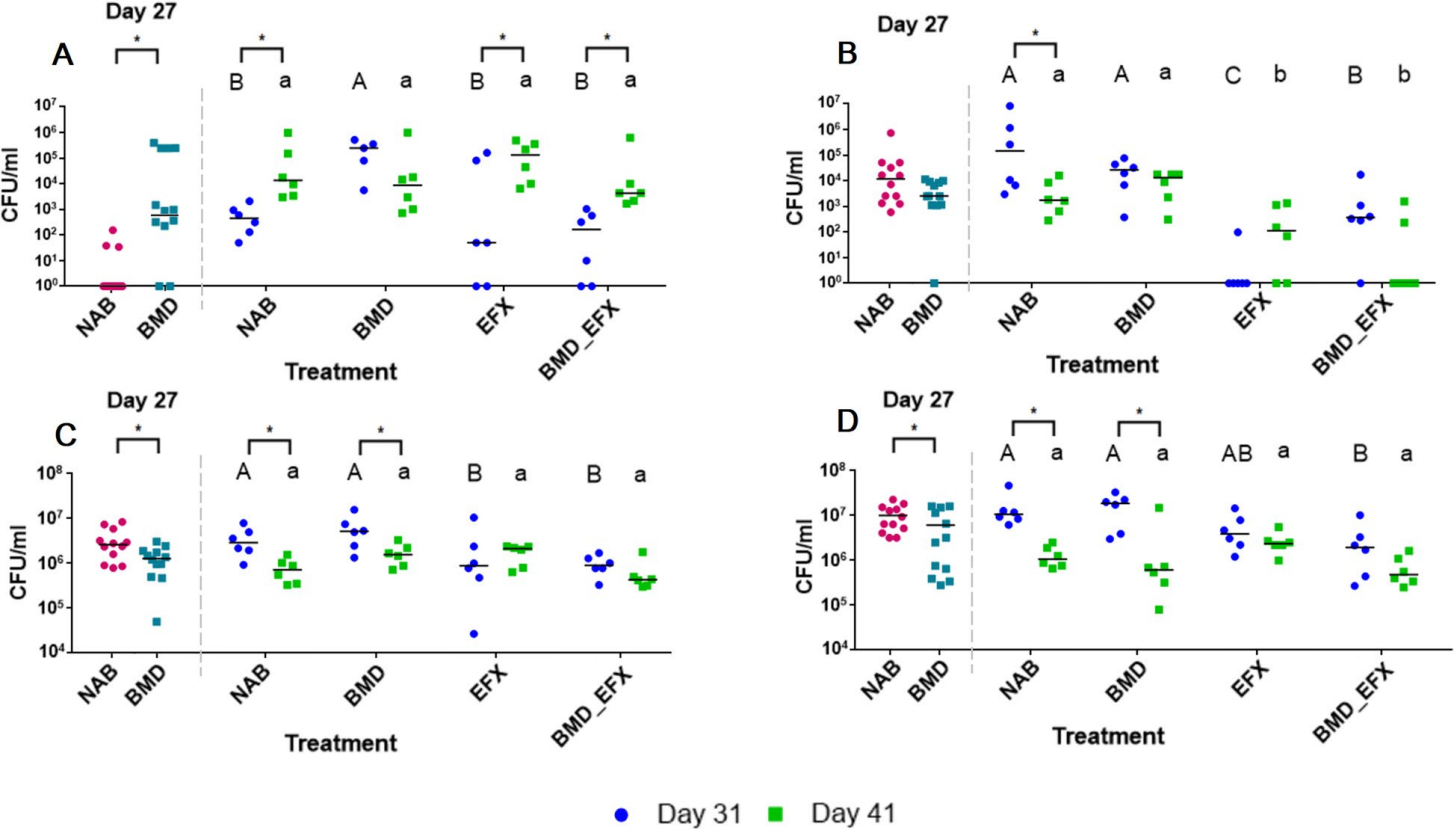
Antibiotic-resistant and respective total counts of targeted bacterial groups in cloacal swabs. In each panel, a dashed line separates data collected at day 27 (two groups, BMD and NAB, *n* = 6 pens and 12 samples each) from data collected at days 31 and 41 (four groups, *n* = 3 pens and 6 samples each). To the left, values marked with asterisks show significant (*p* < 0.05) differences between BMD and NAB. To the right, asterisks show significant (*p* < 0.05) differences between day 31 or 41 within a treatment, upper case letters indicate significant (*p* < 0.05) differences between treatments on day 31, and lower case letters show significant (*p* < 0.05) differences between treatments on day 41. Pairwise comparison of **A** VRE, **B** ESBL, **C** general enterococci, and **D** general coliform counts

**Fig. 6:**
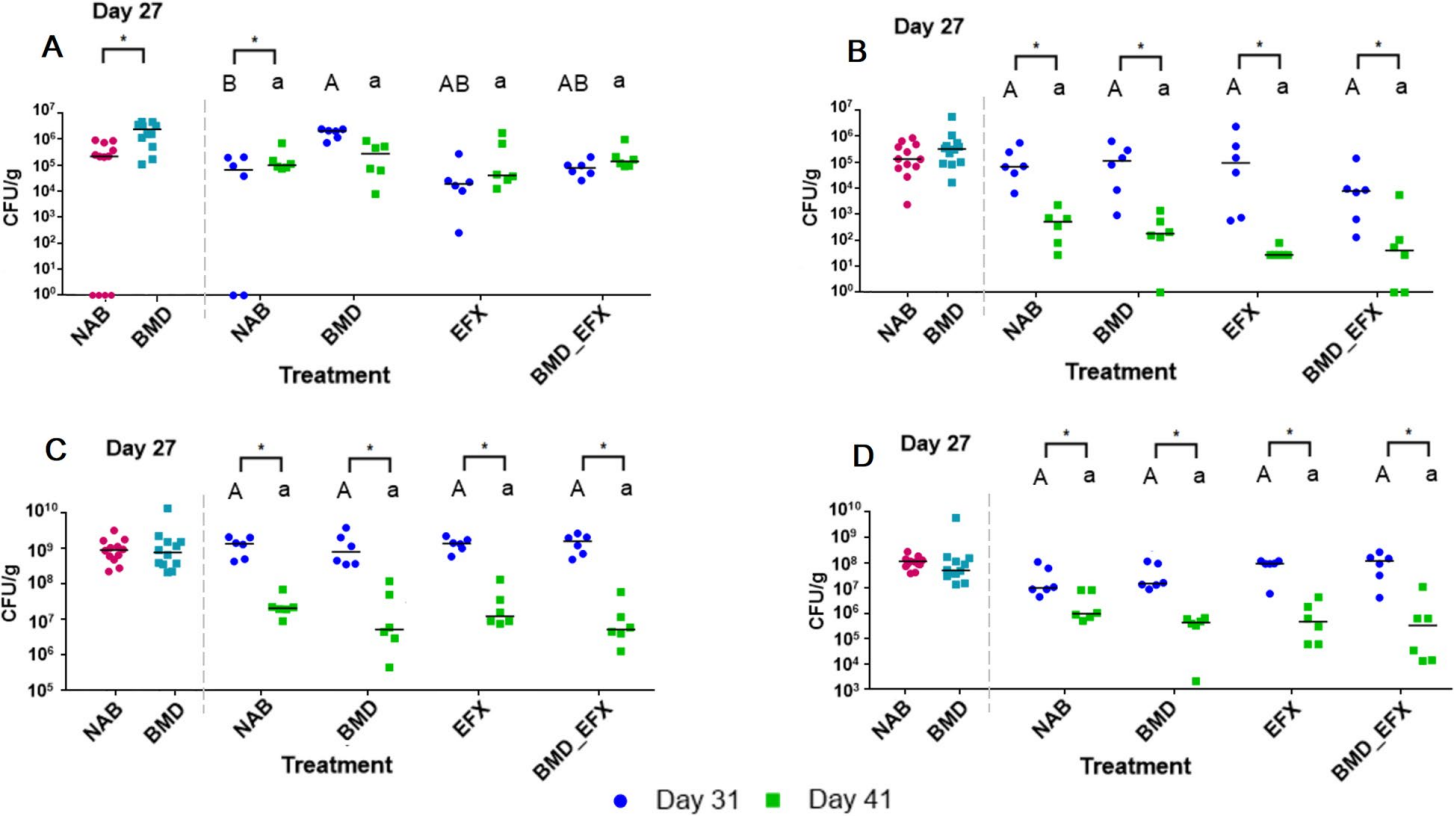
Antibiotic-resistant and respective total counts of targeted bacterial groups in litter samples. In each panel, a dashed line separates data collected at day 27 (two groups, BMD and NAB, *n* = 6 pens and 12 samples each) from data collected at days 31 and 41 (four groups, *n* = 3 pens and 6 samples each). To the left, values marked with asterisks show significant (*p* < 0.05) differences between BMD and NAB. To the right, asterisks show significant (*p* < 0.05) differences between day 31 or 41 within a treatment, upper case letters indicate significant (*p* < 0.05) differences between treatments on day 31, and lower case letters show significant (*p* < 0.05) differences between treatments on day 41. Pairwise comparison of **A** VRE, **B** ESBL, **C** general enterococci, and **D** general coliform counts

Concomitant to the culture-independent molecular analyses, we applied cultivation-based methods to evaluate the presence and relative abundance of methicillin-resistant *Staphylococcus* spp., VRE, and ESBL-E in the cloacal swabs and litter (Figs. 5 and 6) and in meat samples from the same chickens after slaughter. Because general staphylococci counts proved to be unreliable due to the colonies morphological similarity to enterococci, and no methicillin-resistant staphylococci colonies were detected in any of the samples, we decided not to seek other selective methods to screen for staphylococci.

General enterococci counts decreased with time, especially in the litter samples (from about 10^9^ CFU g^−1^ at 27 days and 31 days to 10^7^ at 41 days), and their abundance was not effected by antibiotic treatments. On day 27, VRE-specific counts in BMD-fed chickens relative to the NAB samples were 1–4 log units higher in cloacal swabs, and half to 6 log units higher in the litter. On day 31, BMD treatment resulted in approximately 2 log units higher abundance of VRE relative to NAB in either cloacal swabs or litter. Although higher VRE counts were also observed in EFX-treated chickens, the results were not statistically significant due to high variance. On day 41, average VRE counts in all treatments reached approximately 10^5^–10^6^ CFU mL^−1^, highlighting a temporal increase in VRE abundance regardless of treatment. Total enterococci and VRE-specific counts were higher in the litter samples compared to cloacal swabs by an order of about 3 log units, but the ratio of VRE to total enterococci was similar. Most of the VRE isolates were assigned to avian-associated species (*E. durans n* = 538, *E. gallinarum n* = 150), and, in contrast to metagenomics results, only 14 isolates were identified as *E. faecalis* and one as *E. faecium*. Eighteen (75%) meat samples contained enterococci, ranging from 10^2–3^ CFU g^−1^ regardless of treatment, of which 16 (67%) were VRE. However, all meat VRE isolates were identified as *E. durans*.

A significant decrease in both ESBL-E- specific and general *Enterobacteriaceae* counts was observed in cloacal swabs on day 31, immediately following enrofloxacin treatment (EFX and BMD_EFX groups). Accordingly, ESBL-E counts remained approximately 1 log lower in cloacal swabs from the EFX-treated groups compared to the NAB and BMD-fed groups on day 41. In contrast, enrofloxacin treatment had no impact on ESBL-E counts in the litter, where for all treatment groups, the counts decreased significantly from day 31 to day 41 (from about 10^5^ to 10^2^–10^3^ CFU g^−1^ of litter). Out of 989 ESBL-E isolates, 798 were identified as *E. coli*, 166 as *Klebsiella pneumoniae*, 11 as *Providencia rettgeri*, and one as *Salmonella* spp., *Brevundimonas diminuta*, and *Alcaligenes faecalis* each. All meat samples but one were positive for *Enterobacteriaceae*, ranging from 10^2^ to 10^4^ CFU g^−1^; however, ESBL-E was detected in only one sample at 500 CFU g^−1^.

### Antimicrobial profiling of ESBL-E and VRE isolates

Resistance profiling was performed on selected VRE and ESBL-E isolates (Figures S13, S14, S15 and S16). Among VRE, bacitracin and vancomycin resistance were often correlated and were the main resistance types observed. Streptomycin resistance was observed in cloacal isolates but was rare in the litter. Ampicillin resistance was profuse in isolates from BMD treatments on day 27, but the results were inconsistent thereafter. Collectively, no obvious effect of antibiotic treatment was found in the rate of resistance to single antibiotics in VRE (Figures S13 and S14). Prior to enrofloxacin administration, relatively few ESBL-E colonies were resistant to enrofloxacin, and ciprofloxacin resistance was below 20%. However, on day 31, higher levels of enrofloxacin resistance were observed in the cloacal isolates following EFX administration. This phenomenon was even more pronounced in isolates from the combined BMD_EFX treatment in both cloacal swabs and litter (about 20%, 40%, and 50%). On day 41, enrofloxacin resistance was not observed in cloacal isolates but could still be found in 40% and 20% of litter isolates from the EFX and BMD_EFX treatments, respectively. Ciprofloxacin resistance followed a similar pattern on day 31. Paradoxically, in the cloacal swab, high levels of ciprofloxacin resistance were found in isolates from all treatment groups, with the lowest number in the EFX treatment. Collectively, a transient increase in enrofloxacin resistance was observed in ESBL-E in cloacal samples from the EFX treatment and in litter isolates from the EFX_BMD treatment (Figures S15 and S16).

Isolates were considered MDR if they were resistant to at least three antibiotics. Overall, MDR levels among VRE isolates were low, and they were only detected in cloacal swabs. On day 27, BMD administration resulted in higher numbers of cloacal MDR VRE isolates. On day 31, both antibiotic treatments (BMD and EFX) resulted in higher levels of MDR isolates. On day 41, the abundance of MDR isolates increased in the NAB samples, but an EFX effect was still clearly observed, and almost all isolates from this treatment were MDR (Figures S17 and S18). Most cloacal swab and litter ESBL-E isolates sampled on day 27 were MDR, regardless of treatment. Interestingly, on day 31, cloacal and litter ESBL-E isolates with high MDR levels (resistance to 7–11 antibiotics) were more profuse in EFX treatments when compared to NAB. This phenomenon was also observed in the cloacal isolates from the BMD treatments. Isolates exhibiting high MDR levels increased in the NAB groups on day 41 but remained statistically more prevalent in cloacal isolates from the EFX and EFX_BMD treatments. Overall, treatment with enrofloxacin was correlated to increased levels of MDR, but MDR was also dictated by temporal effects (Figures S19 and S20).

## Discussion

Numerous studies have evaluated the scope and diversity of antimicrobial resistance in poultry facilities using traditional culture-based methods [27–30], and over the past decade, metagenomic approaches, facilitated by breakthroughs in next-generation sequencing and bioinformatics, have significantly contributed to the exploration of AMR in complex microbial communities [31, 32]. While both of these approaches have specific advantages, when performed individually, they often provide an incomplete picture of antibiotic resistance in complex ecosystems. To the best of our knowledge, this comprehensive longitudinal study is the first to assess AMR in animal husbandry facilities by complementing metagenomic approaches with culture-based analyses that specifically targeted AMR in priority pathogens. It provided four principal ecological and public heath insights that substantially enhance our capacity to acquire a “one health” perspective on AMR dynamics in broiler chicken facilities: (i) cloaca swab and litter microbiomes and resistomes are substantially different, and thus the first may be targeted for source tracking of AMR and pathogens in meat and within poultry farms, whereas the later may provide insight into the transmission pathways in the environment (i.e., litter fertilized crops and runoff); (ii) fecal and litter microbiomes and resistomes are strongly dictated by temporal dynamics and appear to become more diverse and stable with time with a decrease in priority pathogen-associated phyla but an apparent increase in the resistance of these strains; (iii) from a global “metagenomic” perspective, growth-promoting (bacitracin) and therapeutic (enrofloxacin) antibiotics do not substantially influence cloacal and litter microbiomes and resistomes, but these antibiotics can facilitate resistance in specific bacteria (including potential pathogens) at specific points during chicken development, especially in the gut; and (iv) of the priority pathogens, *E. faecium*, *E. faecalis*, *E. coli*, and *C. jejuni* appear to be the most clinically relevant bacteria species in chicken cloaca and litter. The latter two species appear to be the most hazardous considering their virulence and scope of ARGs.

### Inferring the most relevant AMR priority pathogens in chicken cloaca and litter

Using our integrated culture-based and culture-independent approach, we targeted *Enterobacteriaceae*, *Enterococcaceae*, and *Staphylococcaceae*, specifically focusing on strains resistance to third-generation cephalosporins, vancomycin, and methicillin, respectively, because these ARB are of major public health concern and are considered to be potential drivers of AMR from zoonotic to human pathogens [33, 34]. We also targeted *Campylobacteraceae* following the detection of a MAG characterized as *C. jejuni* that harbored multiple ARGs that confer resistance to fluoroquinolones, β-lactams, bacitracin, and macrolides, considering the role of this zoonotic pathogen in poultry associated food poisoning [35]. Similar MDR *C. jejuni* strains have been indicated in clinical outbreaks worldwide [36]. *S. aureus* was not detected by either culture-based or metagenomic analyses, suggesting that this group was not a significant factor in the targeted facility; this was complemented by the extremely low abundance of *mec*A, a gene frequently associated with human MRSA [37, 38].

Culture-based and shotgun metagenomic analyses detected high levels of total and vancomycin-resistant *Enterococcaceae* in both cloacal swabs and litter. Taxonomic extrapolation of enterococci from metagenomes revealed that depending on sampling time and treatment, between 28 and 76% were human pathogen related (i.e., *E. faecalis* and *E. faecium*). In contrast, MALDI-TOF–based screening revealed that the vast majority of VRE isolates were typical avian-associated strains (*E. durans* and *E. gallinarum*), and ~ 2% were classified as *E. faecalis* or *E. faecium*. While this may stem from errors in metagenomic annotation, we hypothesize that this discrepancy stems from the fact that although the commensal pathogens *E. faecalis* and *E. faecium* are profuse in poultry litter and cloacal swabs, they are predominantly sensitive to vancomycin. This is supported by annotation of *Enterococcaceae* MAGs, which revealed that *E. faecalis* and *E. faecium* genomes lacked vancomycin resistance genes in contrast to the presence of genes (e.g., *vanC*, *vanSC*, *vanRC*, and *vanTC*) in some of the non-pathogenic *E. gallinarum* and *E. casseliflavus* genomes, and by the extremely low abundance of *van*A in metagenomic samples, which is traditionally linked to resistance in *E. faecalis* and *E. faecium* [39].

Isolation and metagenomic analyses revealed that the vast majority (~ 86% collectively of cloacal swabs and litter) of total *Enterobacteriaceae* were *E. coli*. MAG screening and network analysis revealed that *Enterobacteriaceae* harbored most of the ARGs characterized in this study, undoubtedly due to the strong bias of current ARG databases towards *E. coli* [31]. *Enterobacteriaceae*-associated ARGs were predominantly multidrug efflux pumps, and almost no β-lactamases were found to be associated with this group. However, in *E. coli*, these genes, and especially ESBL- and carbapenemase-encoding genes, are frequently harbored on plasmids, and thus not on MAGs, and not linked to specific taxa due to horizontal transfer. Nonetheless, specific ESBL-conferring β-lactamases (i.e., *bla*SHV, *bla*TEM, and *bla*OXA-1) were identified in the metagenomic analysis. These genes are frequently harbored on multidrug-resistant plasmids, suggesting the presence of such plasmids in the identified ESBL isolates [40]. Carbapenemase genes were not detected in any of the samples, correlating to the almost complete absence of carbapenem resistance in screened ESBL isolates, suggesting that resistance to this “last resort” antibiotic has not substantially emerged in the targeted broiler facility. In contrast, a relatively large fraction of ESBL-E were resistant to the last resort antibiotic colistin, although plasmid-associated colistin resistant genes (i.e., *mcr-1*) were not detected in the metagenomic analyses. This may be due to intrinsic resistance to colistin or the presence of currently uncharacterized resistance genes [41].

### Bacitracin and enrofloxacin select for specific bacterial phyla and ARGs

In early growth stages, bacitracin-fed chickens contained substantially higher levels of VRE than chickens not fed growth promoters. This may be attributed to the presence of *upp*P, detected in the *E. galinarium*, *E. feacalis*, *E. faecium*, and *E. casseliflavus* MAGs, which was previously documented to confer low-level bacitracin resistance in *E. faecalis* [42]. BMD feeding also resulted in the increased relative abundance of *Selenomonadaceae*, a family previously determined to be transmitted from adult hens to the caecal microbiota of newly hatched chicks [43]. Metagenomic analysis revealed several bacitracin resistance genes (e.g., *bcrA, bcrD*, and *uppP*), which at certain time points (most notably day 31) were higher in BMD-fed chickens, suggesting a selection of resistant strains. However, the correlation between BMD and the relative abundance of bacitracin resistance genes on day 41 was not maintained in the litter, and HT-qPCR showed a lower relative abundance of the bacitracin resistance gene *bacA-02* at this time, implying that the dynamics of bacitracin resistance are complex and dependent on multiple factors.

Enrofloxacin had varying effects on swab and litter microbiomes and resistomes when collectively analyzing culture-based and culture-independent data. The relative abundance of quinolone resistance genes in swabs increased following the application of enrofloxacin, but the opposite effect was observed in the litter. This suggests that enrofloxacin concentrations facilitate selection in the chicken gut but are not high enough to cause selection in the litter. Interestingly in the litter, both antibiotic treatments resulted in the increased relative abundance of the *E. coli*-associated multidrug efflux pump-encoding gene *emrD* [44–46]. While we cannot specifically determine which resistances are conferred by this efflux pump, its increased abundance may be associated with the fact that more MDR ESBL-E were observed in cultures from enrofloxacin-treated groups. On day 41, both BMD and EFX treatments resulted in the increased relative abundance of the class 2 integron integrase gene *int*2. This gene is strongly associated with plasmids and with a myriad of ARGs including the tetracycline resistance gene, *tetX* [47, 48]. Interestingly, both *tetX* and *int*2 genes increased in the litter of the combination (BMD_EFX) treatment on day 41. Previous experiments indicated that class 2 integrons play an important role in the prevalence of ARGs in chicken litter and in facilitating gene transfer to downstream microbiomes [47] and that they are associated with plasmids that harbor ESBL-encoding genes [49].

## Conclusions

Our results demonstrate the high complexity of poultry microbiomes and resistomes and the fact that the gut and litter microbiomes and their associated ARGs mature and stabilize with time. Administration of antibiotics (whether at therapeutic or sub-therapeutic doses) affects microbial communities and associated ARGs in a highly complex manner, and therefore, it is not possible to visualize their effect on AMR as simplistic cause and effect relationships. More longitudinal studies like this one are required to determine baseline microbiomes and resistome levels in broiler production facilities and to evaluate the effect of different antibiotic classes. Nonetheless, the evidence indicating BMD stimulates VRE, and the presence of *C. jejuni* harboring fluoroquinolone and β-lactamase genes in cloacal swabs from enrofloxacin-treated chickens provides direct evidence of risk, and therefore, the use of these antibiotics needs to be strongly regulated.

## Methods

### Experimental design

The study was conducted between May and June 2018 in an experimental poultry house, simulating a full commercial broiler production cycle. The poultry house was divided into 12 pens with poultry mesh, with six pens on each side separated by a 2-m–wide service lane. The house was empty for about a year before the experiment and was free of animal and feed residues. After thorough cleaning and disinfection, each pen was covered with fresh wood shavings and supplied with a hanging hopper feeder and an automatic water dispenser. Four hundred-one-day-old male broiler Ross 308 chicks were obtained from a breeder flock of hens, during their optimal period of egg production (37 weeks old). The chicks were individually weighted, and 360 chicks with a body weight (BW) of 37 ± 1.5 g were selected. Each chick was individually tagged and divided according to their BW into 2 dietary [BMD-supplemented (using a standard AGP dose of 55 ppm) or non-supplemented feed] treatments (*n* = 180 per group). Each dietary treatment was divided into 6 litter pens, with 30 animals per pen for a density of 0.07 m^2^/bird. On day 28, half of the animals in each dietary treatment group (with and without BMD, 3 pens out of the six) were treated for 3 days with the fluoroquinolone enrofloxacin (EFX), administered through drinking water at standard therapeutic concentrations of 10 mg kg^−1^ (50 ppm). Enrofloxacin was chosen because it is one of the most used antibiotics for the treatment of a variety of infections in poultry production in Israel. In summary, the experiment consisted of four treatments: (I) no-antibiotic (NAB), (II) BMD, (III) EFX, and (IV) BMD_EFX. Dead birds were removed, and date and body weight were recorded at removal.

Birds were weighed weekly, and weekly food intake per pen was calculated. All procedures were performed with biosafety measures to prevent cross-contamination between pens, such as exchange of gloves and overshoes. At the end of the experiment, at the age of 41 days, the chickens were individually weighted and the feed was removed for 12 h before slaughter. The breast muscle, abdominal fat pad, heart, and liver were removed and weighed, and their weights calculated relative to their live body weight.

### Sampling and sample processing

Cloacal swabs and environmental samples (litter) were sampled in each pen on day 27 (prior to EFX treatment), day 31 (immediately after EFX treatment), and day 41 (at the end of the growth cycle). In addition, meat (internal pectoral muscle) was sampled after slaughter. The study was longitudinal, wherein cloacal swabs from the same animals were repeatedly sampled in order to increase efficacy. On day 27, the first sampling, ten chickens in each yard were randomly selected for fecal swab sampling and their numbers recorded. Cloacal swabs were subsequently sampled from these same animals on days 31 and 41, and dead animals were not replaced in the next samplings. For environmental sampling, litter samples were taken from at least five different random spots in each pen with a sterile plastic bag and carefully mixed. Samples were kept cooled and transferred to the laboratory within no more than 3 h.

In the laboratory, five cloacal swabs from the same pen were combined into one composite sample. One replicate was used for the isolation of bacteria and the other for DNA extraction for subsequent molecular analyses (see below). Swab tips were cut with sterile scissors (exchanged between composite samples) into 4 mL sterile phosphate-buffered saline (PBS) in 15-mL conical cork tubes and vortexed at maximum speed for 10 min. The suspension was transferred to a new sterile test tube. For environmental sampling, two 5-g litter samples were separately suspended into 20 mL of PBS per pen. Replicate samples were vortexed at maximum speed for 5 min to achieve a homogeneous mixture and then centrifuged at 500 RPM for 15 min to sink bulk material. A volume of 5 mL of supernatant was then transferred to a new sterile tube.

Meat samples were collected from the same animals sampled throughout the study after slaughter. The whole breast meat was brought to the laboratory and externally disinfected by burning to minimize the risk of cross-contamination. Then, 1-g samples of the internal pectoral muscles from each side of each animal were collected and pooled according to the same groups of five animals sampled together by cloacal swabs in each pen. Thus, two composite samples of 10-g meat were obtained per pen. Meat samples were homogenized in a sterile stomacher bag containing filter mash with 90 mL of PBS for 10 min. The filtered chicken meat suspension was subjected to two-stage centrifugation: low-speed centrifugation (1000 RPM, 15 min) to precipitate meat residues, followed by high-speed centrifugation (5000 RPM, 15 min) to pellet bacteria. The bacterial pellet was suspended in 4 mL PBS. For bacterial culturing, pellets were suspended in 30% glycerol PBS (see below).

### DNA extraction

DNA was extracted from pelleted composite cloacal swab samples or directly from composite litter samples using the GenALL DNA extraction kit (GeneAll Biotechnology Co. Ltd., Seoul, South Korea), using the protocol provided by the manufacturer. DNA concentration and quality were determined using a NanoDrop 2000c Spectrophotometer (Thermo Fisher Scientific, Wilmington, DE., USA) and a Qbit Fluorimeter (Invitrogen, Carlsbad, CA, USA) and validated by gel electrophoresis.

### Shotgun metagenome sequencing and analysis

#### Library preparation, shotgun metagenome sequencing, and assembly

Library preparation and metagenomic sequencing of all 21 samples (*n* = 11 cloacal swabs and *n* = 10 litter samples) were performed at the University of Illinois at Chicago DNA Services Facility, USA. Sequencing was performed on the Illumina Novaseq platform with the sequencing strategy of 150-bp paired-end reads. Read quality was evaluated by the FastQC tool [50] and adjusted by Trimmomatic software [51]. Chicken DNA was removed by mapping of quality-filtered reads to the chicken genome (NCBI Genome ID: 111; GRCg6a) employing Bowtie2 [52] and SAM tools [53]. Subsequently, de novo genome assembly of processed reads from all of the samples was achieved with metaSPAdes [54], utilizing a *k*-mer range of 21–55 to obtain the maximum number of contigs and the maximum value of N50 [55].

#### Open reading frame (ORF) prediction and annotation

Open reading frame (ORF) prediction was performed on scaffolds utilizing Prodigal [56]. To obtain a non-redundant ORF set for poultry samples, we used CD-HIT [57] with a 95% identity threshold. Finally, reads were mapped back to this non-redundant ORF set for each sample using Bowtie2, and the coverage for each ORF was calculated as the number of mapped reads. For the taxonomic classification, we mapped representative ORF set against the NCBI-NR protein database and the analysis of generated output was performed by MEGAN [58] using the lowest common ancestor algorithm and default parameters. To characterize ARGs, we employed the DeepARGs database that consists of more than 14,000 non-redundant ARG sequences from CARD, ARDB, and UniProt databases [59]. For this study, mapping was performed using Diamond [60] in sensitive mode, with a BLASTX E-value cutoff of 1e^−5^ and an identity and query coverage threshold of 70% to condense false-positive predictions [61–63]. Finally, a custom python script was used to attain the final count/abundance data of the annotated terms.

#### Metagenome-assembled genome (MAG) reconstruction

MAGs were reconstructed using the following pipeline. Initially, metaSPAdes-generated scaffolds were further indexed, and short reads were mapped back using bowtie2 (v2.3.5.1). Samtools (v1.9) was used to sort the read mappings, and the read coverage was calculated using the MetaBAT2 script (jgi_summarize_bam_contig_depths). We then identified MAGs (putative member bins) using MetaBAT2 (v2.10.2) [64], after filtering scaffolds at least 5000 bp in length. For each identified bin, we performed genome quality estimation using CheckM (v1.1.2) [65] and annotation employing Prokka (v1.14.6) [66]. The dRep (v2.3.2) [67] workflow was used for de-replication of the entire set of MAGs and to obtain representative putative genomes. Finally, taxonomic annotation of recovered MAGs was performed with GTDB-Tk (v1.5.0) [68] using the “classify_wf” function and default parameters.

### 16S rRNA gene amplicon sequencing and analysis

The litter microbiota was further profiled by sequencing the V4 region of the 16S rRNA gene for all DNA samples (*n* = 30 from 10 chicken litter samples in triplicates) and negative controls (*n* = 3). Extracted litter DNA (see above) was amplified using the CS1-515F and CS2-806R primer set to generate sequencer-ready libraries as previously described [69, 70]. The barcoded libraries were pooled and sequenced on an Illumina MiSeq platform (with a strategy of 300-bp paired-end) employing V3 chemistry for 16S genes at the University of Illinois at Chicago DNA Services Facility. Around 1.05 GB of sequencing data was generated with an average of approx. 91,000 sequences per sample excluding negative controls (approx. 700 sequences per sample). Generated paired-end sequences were processed using Qiime2 v2018.11 [71] after primer sequences were removed using the cutadapt tool [72]. The obtained sequences underwent quality filtering employing the DADA2 algorithm [73], which resolves amplicon sequence errors to generate amplicon sequence variants (ASVs). Taxonomic assignments were performed using a Naive Bayes classifier trained on the SILVA 132 rRNA database, using a 99% OTU cutoff [74]. Additionally, samples with low sequencing depth such as negative controls (< 1000 reads) and ASVs without any reads in any of the samples were excluded from further analysis.

### HT-qPCR

Concomitant to 16S rRNA amplicon sequencing, we analyzed the composition and relative abundance of selected ARGs and MGEs in litter samples using a custom HT-qPCR array, specifically investigating 53 clinically relevant ARGs and MGEs (mentioned in Table S7) found to be abundant or present in the metagenomic analysis. Litter DNA (*n* = 30 from 10 chicken litter samples in triplicates) and negative controls (*n* = 3) were transferred to the Key Lab of Urban Environment and Health, Institute of Urban Environment, Chinese Academy of Sciences (Xiamen, China), where HT-qPCR was performed using the Wafergen Smart Chip Real-time PCR system as previously described [75].

### Network analysis

Network analysis was performed to reveal the underlying associations between ARGs, MGEs (annotated with INTEGRALL—http://integrall.bio.ua.pt/), and microbial taxa. A correlation matrix was constructed by calculating all possible pairwise Spearman correlation coefficients (rho) among the ARG subtypes and bacterial families, ARG subtypes and MGEs, and MGEs and bacterial families based on the normalized abundance data obtained from the metagenomic analysis. ARGs and MGEs present in at least one-third of the samples (7 out of 21 chicken samples) were included for network correlation analysis. Only statistically strong (rho > 0.80) and significant correlations (padj < 0.01) were kept for this study [63, 76]. Finally, the Gephi software package (v0.9.2) was used to visualize the correlation network [77].

### Microbiological isolation and characterization

#### Bacterial counts

Serial decimal dilutions were performed from sample suspensions (swabs, litter, and meat) on the day of sampling. From each dilution, 100 mL were inoculated onto agar plates (see below) and spread with a disposable Drigalski spatula until dry. Three bacterial groups were investigated due to their importance as resistance indicators and in public health: *Enterobacteriaceae*, staphylococci, and enterococci. For “general” population counts, the following media were used: MacConkey for *Enterobacteriaceae* (coliforms), KF for enterococci, and Baird-Parker for staphylococci. For the major corresponding resistant bacteria variants, the following selective media were used respectively (all purchased as ready-to-use plates from Hylabs, Rehovot, Israel): CHROMagar ESBL, CHROMagar VRE, and CHROMagar MRSA. Plates were incubated overnight at 37 °C, except for VRE plates that were incubated for 48 h. After incubation, colonies were counted using morphological criteria. On MacConkey plates, only red (lactase-positive) colonies corresponding to coliforms (mainly *E. coli*, *Klebsiella* spp., *Enterobacter* spp., *Citrobacter* spp.) were counted. The same was done with KF and Baird-Parker agars, counting only enterococci and staphylococci corresponding colonies. With CHROMagar plates, only target colonies were counted, as by the manufacturer guides. Prior to the beginning of the experiment, preliminary studies were performed using poultry cloacal swabs and environmental samples to set the most adequate range of dilutions to be plated. In these preliminary experiments, the use of Tween 80 as an emulsifier to release bacteria in litter samples was also tested. Tween 80 did not improve bacterial counts, and therefore, it was opted not to use an emulsifier in litter sample processing. It was also found that the number of bacteria and amount of DNA retrieved from individual cloacal swabs were too low, thus precluding processing of swabs individually. Also prior to the experiment, two members of the research group were trained on the different colony morphologies in all the agar types used by streaking known isolates of Enterobacteriaceae, enterococcus, and staphylococcus species on the respective media. For better consistency, one of these two research members was assigned to do all the bacterial counting under the supervision of the second trained research member. Colony counts were performed under a magnifying glass and recorded. The results were expressed as CFU g^−1^ (litter, meat) or mL (cloacal swab solution), depending on the sample type. For validation, random target colonies with different morphologies were selected and identified by MALDI-TOF mass spectrometry (see below).

#### Bacterial isolation, identification, and culture maintenance

For each sampling time and treatment group, 24 representative VRE and ESBL colonies (approximately eight colonies per pen) were picked and streaked on blood agar (tryptose blood agar base enriched with 5% sheep washed blood cells). Bacterial identification was performed by MALDI-TOF mass spectrometry with an Autoflex II mass spectrometer (Bruker Daltonics, Billerica, MA, USA) using the manufacturer’s instructions, with either the direct (ESBL-E) or on-target formic acid treatment (VRE) protocols. Data were automatically acquired using the Flex control 3.0 and MaldiBiotyper Automation Control 2.0. software (Bruker Daltonics GmbH, Bremen, Germany) following the standard microbial identification method and using the MBT 7311 species database. Scores over 2.0 were considered for species-level identification. VRE colonies were further screened by sequencing the *tuf* gene as previously described [78]. Identified colonies were preserved in deep-well plates in Brain–Heart Infusion broth (BHI 37%, Difco) with 30% glycerol at − 80 °C.

#### Antimicrobial susceptibility testing

Resistance of stocked isolates to additional antibiotics was evaluated using the broth microdilution assay following Clinical Laboratory Standard Institute instructions. End-point concentrations were chosen for each antimicrobial drug tested, using clinical resistance breakpoints [79]. All antimicrobials were purchased from Sigma, Germany. Preserved ESBL-E and VRE isolates were inoculated into 200 µl Cation-adjusted Mueller–Hinton broth (CAMHB) in a sterile 96-well plate and incubated overnight at 37 °C. Then, bacteria were inoculated into CAMHB in a new 96-well plate using a sterilized plate replicator and incubated for 6 h for log-phase growth. After the second incubation, bacteria were inoculated with a sterilized plate replicator into CAMHB supplemented with the below-described antimicrobials (one per plate) and incubated overnight at 37 °C. Growth (resistance) was assessed by the turbidity of the growth medium. ESBL-E were tested for resistance to the following eleven antimicrobials at the following concentrations: ampicillin (AMP, 32 µg/mL), cefotaxime (CTX, 4 µg/mL),meropenen (MEM, 16 µg/mL), enrofloxacin (EFX, 64 µg/mL), ciprofloxacin (CIP, 4 µg/mL), gentamycin (GEN, 16 µg/mL), kanamycin (KAN, 64 µg/mL), chloramphenicol (CHL, 32 µg/mL), colistin (CST, 8 µg/mL), tetracycline (TET, 16 µg/mL), and bacitracin (BAC, 64 µg/mL). VRE were tested for resistance to the following five antimicrobials at the following concentrations: vancomycin (VAN, 32 µg/mL), gentamycin (GEN, 16 µg/mL), streptomycin (STR, 64 µg/mL), ampicillin (AMP, 32 µg/mL), and bacitracin (BAC, 64 µg/mL). For multidrug resistance (MDR) comparison, VRE isolates were classified into non-MDR (resistance to 0–2 antimicrobials), low-level MDR (3 antimicrobials), intermediate-level MDR (4 antimicrobials), or high-level MDR (5 antimicrobials) and ESBL-E isolates into non-MDR (0–2 antimicrobials), low (3–4 antimicrobials), intermediate (5–6 antimicrobials), and high (> 7 antimicrobials).

### Statistical analyses

Animal growth parameters were analyzed as follows. The effects of the two feed types (with or without BMD) on body weight from 0 to 27 days (two groups, before enrofloxacin treatment) were assessed using the following ANOVA mixed model:

$$Y=\mu + dietary \; treatment+\; pen \; \left[ dietary \; treatment\right]+e$$

with dietary treatment (BMD-supplemented feed vs. feed without antibiotics) as the main fixed effect and pen as a random nested effect. For data after 31 days (after enrofloxacin treatment), the treatment effects (BMD, EFX, BMD_EFX) on body weight as well as meat processing parameters were assessed using the following two-way ANOVA mix model:

$$Y=\mu + dietary \; treatment+ EFX+ dietary \; treatment\times EFX+ pen \; \left[ dietary \; treatment\times EFX\right]+e$$

with treatments as the main fixed effects and all their interactions and pen as a random nested effect. The Tukey–Kramer HSD test was used for post hoc testing of the differences between the LS means of the four treatments. The analysis of mortality as the proportion of “1” dead vs. “0” alive individuals within each of the four treatments was tested by the chi-square test.

For the bacteriological statistics, colony count data were log transformed, and analysis was performed separately for cloacal swab and litter results. BMD-supplemented and non-supplemented bacterial counts (*n* = 12 each) at day 27 (first sampling) were compared by the two-sample *t*-test. For data from sampling at days 31 and 41, repeated measures ANOVA-fixed effects were determined for the treatment combinations of BMD and EFX (four treatments: NAB, EFX, BMD, BMD_EFX, *n* = 6 each) and day (31 or 41). For pre-analysis, all data underwent a square root transformation to normalize and stabilize variances. Sample (replicates within the same pen, *n* = 6 per treatment) nested within treatment combination was a random factor. When the interaction between treatment combination and day was significant, post hoc comparisons of treatment combinations were performed for each day by contrast *t*-test with Bonferroni correction (*p* = 0.05). Differences in MDR patterns between treatments and sampling day were tested by Pearson’s *χ*^2^ test using the JMP 15 software.

For metagenomic data, alpha‐diversity matrices such as Shannon Diversity Index were used to measure the diversity within samples for chicken litter obtained from amplicon sequencing data analysis. The Kruskal–Wallis test followed by multiple pairwise comparison using Dunn’s post hoc method was applied to the alpha‐diversity metrics to assess the statistically significant differences in the diversity within samples between the groups. Beta-diversity analysis was performed to see the relationship between samples and was visualized with non-metric multidimensional scaling (NMDS) plots of Bray–Curtis distance metrics using the vegan package in R [80]. The DESeq2 package [81] was used for the analysis of differentially abundant bacteria (from amplicon data) and/or ARGs (from HT-qPCR array data) that are statistically enriched or depleted in particular treatments and/or time points of chicken litter samples by calculating fold change values. Moreover, Friedman’s test followed by multiple pairwise comparison using the Nemenyi post hoc test using the PMCMR package in R was employed to assess the statistical significance of three pathogenic families (*Enterobacteriaceae*, *Enterococcaceae*, and *Staphylococcaceae*) over treatments and/or time points in chicken litter samples obtained from amplicon data analysis.

## Supplementary Information

**Additional file 1: Figure S1.** Temporal increase in body weight of broiler chickens for the duration of the growth cycle. Left side, chickens fed with or without BMD (NAB) up to day 27. Right side, body weight immediately after enrofloxacin treatment (EFX and BMD_EFX) on day 41. **Figure S2.** Individual production parameters: body weight on day of slaughter and meat processing parameters (relative breast, heart, liver and abdominal fat weights) after slaughter of chicken in the four different treatment types. a-b indicate statistical significant differences (*p* < 0.05). **Figure S3.** Distribution of microbial domains in cloacal swab and litter (A); and the relative abundance of prominent non-bacterial (Archaea, Eukaryota and Viruses) families (B) as a function of sampling time and treatment. The abundance data was normalized by scaling each row separately to emphasize abundance as a function of treatment. **Figure S4.** Diversity and bacterial community composition in litter derived from 16S rRNA gene amplicon sequencing. (A) Temporal fluctuations in alpha diversity (Shannon index). Values are presented as the median (black horizontal line), lower and upper hinges correspond to the 25th and 75th percentiles and the outliers are displayed by small black dots; and (B) relative abundance of bacterial families; unclassified taxa and bacterial families having relative abundance less than 0.5% were grouped into “Others”. The relative abundance of each family was represented as the mean value of the biological triplicates of corresponding samples. *p < 0.05, and n.s. indicates *p* > 0.05 by Kruskal-Wallis test followed by pairwise comparison using Dunn’s post-hoc method. **Figure S5.** Temporal changes in the distribution of the three-targeted pathogenic-associated bacterial families: (A) Enterobacteriaceae, (B) Entercococaceae, and (C) Staphylococaceae, in litter derived from 16S rRNA gene amplicon sequencing data. The relative abundance values are presented as the median (black horizontal line), lower and upper hinges corresponds to the 25th and 75th percentiles. The outliers are displayed by small black dots. *p < 0.05, **p < 0.01, and n.s. indicates *p* > 0.05, by Friedman’s test followed by pairwise comparison using Nemenyi pot-hoc test. **Figure S6.** A. Differentially abundant bacterial genera (derived from 16S rRNA gene amplicon sequencing) observed in chicken litter over the time. The Log2Fold change value of bacterial genus at padj (adjusted *p*-value) <0.10 are presented here and significant Log2Fold change with padj <0.05 and <0.01 were marked by single and double stars respectively. The red and blue boxes represents enriched and suppressed genera over the time. Bacterial genus are also grouped by their family and phylum. B. Litter-associated bacterial genera positively or negatively correlated to antibiotic treatments derived from 16S rRNA gene amplicon sequencing. Significant Log2Fold changes (antibiotic treatment relative to corresponding NAB samples) with padj <0.05 and <0.01 are marked by single and double stars respectively. Red and blue boxes represent antibiotic-stimulated and antibiotic-suppressed genera, respectively. **Figure S7.** Relative distribution of bacterial species within (A) Enterobacteriaceae, (B) Staphylococcaceae, and (C) Enterococcaceae families in the cloacal swab and litter metagenomes. **Figure S8.** Dendrogram generated from MASH analysis (dRep workflow) of potential pathogen-associated family MAGs recovered from (A) cloacal swabs (*n* = 38) and (B) litter (*n* = 19). The de-replication analysis suggested a reduced set of 17 and 7 representative MAGs for swab and litter, respectively. **Figure S9.** Priority (Rank-I) ARGs in cloacal swab and litter samples based on ARG-ranker (https://github.com/caozhichongchong/arg_ranker) predictions. **Figure S10.** Relative abundance of ARGs and MGEs in chicken litter samples obtained from HTqPCR (High-throughput quantitate PCR) data analysis. The mean abundance of triplicate biological samples was normalized by scaling each row separately to better visualize the impact of individual treatments. **Figure S11.** A. Differentially abundant ARGs and MGEs (derived from HT-qPCR analysis) observed in chicken litter over time. The Log2Fold change of ARG/MGE values with padj (adjusted *p*-value) <0.10 are presented, and significant Log2Fold change with padj <0.05 and <0.01 are marked by single and double stars respectively. The red and blue boxes represent ARGs/MGEs that significantly increase and decrease over the time. ARGs/MGEs are also grouped by category. B. Litter-associated ARGs and MGEs positively or negatively correlated to antibiotic treatments derived from HT-qPCR analysis. Significant Log2Fold changes (antibiotic treatment relative to corresponding NAB samples) with. padj <0.05 and <0.01 are marked by single and double stars respectively. Red and blue boxes represent antibiotic-stimulated and antibiotic-suppressed ARGs/MGEs, respectively. **Figure S12.** Network analysis showing correlations between (A) ARGs and MGEs, and (B) MGEs and bacterial families. Nodes show ARGs, MGEs and bacterial families and edges (i.e., connections between ARG and MGE, or MGE and family) indicate significant (padj <0.01) and strong pairwise correlations (spearman rho >0.80). The size of each node is proportional to the number of connections (i.e. degree) and the edge thickness is proportional to the spearman correlation coefficient (rho; 0.80–0.99). MLS: Macrolide-Lincosamide-Streptogramin; MGEs: mobile genetic elements. **Figure S13.** Relative abundance (%) of VRE isolates from day 27 resistant to additional antibiotics. Cloacal swabs: 27S_NAB (*n* = 48), 27S_BMD (*n* = 78). Litter: 27L_NAB (*n* = 34), 27L_BMD (*n* = 48). **Figure S14.** Relative abundance (%) of VRE isolates resistant to additional antibiotics from (A) cloacal swabs and (B) litter. Cloacal swabs: NAB (31d *n* = 48, 41d *n* = 47), BMD (31d *n* = 37, 41d *n* = 48), EFX (31d *n* = 48, 41d *n* = 31), BMD_EFX (31d *n* = 40, 41d *n* = 72). Litter: NAB (31d *n* = 17, 41d *n* = 28), BMD (31d *n* = 23, 41d *n* = 31), EFX (31d *n* = 36, 41d *n* = 24), BMD_EFX (31d *n* = 29, 41d *n* = 20). **Figure S15.** Relative abundance (%) of ESBL-E isolates resistant to additional antibiotics isolated on day 27. Cloacal swabs: 27S_NAB (*n* = 113), 27S_BMD (*n* = 96). Litter: 27L_NAB (*n* = 155), 27L_BMD (*n* = 113). **Figure S16.** Relative abundance (%) of ESBL-E isolates resistant to additional antibiotics from (A) cloacal swabs and (B) litter. Cloacal swabs: NAB (31d *n* = 56, 41d *n* = 48), BMD (31d *n* = 48, 41d *n* = 40), EFX (31d *n* = 2, 41d *n* = 34), BMD_EFX (31d *n* = 32, 41d *n* = 33). Litter: NAB (31d *n* = 36, 41d *n* = 21), BMD (31d *n* = 34, 41d *n* = 24), EFX (31d *n* = 45, 41d *n* = 10), BMD_EFX (31d *n* = 29, 41d *n* = 20). **Figure S17.** Relative abundance (%) of multidrug resistant VRE isolates isolated on day 27, resistant to 0-2, 3, 4 and 5-6 different antibiotics. Diagonal lines represent a significant difference compared to the control (NAB) on the same day. Cloacal swabs: 27S_NAB (*n* = 48), 27S_BMD (*n* = 78). Litter: 27L_NAB (*n* = 34), 27L_BMD (*n* = 48). **Figure S18.** Relative abundance (%) of multidrug resistant VRE isolates from days 31 and 41, resistant to 0-2, 3, 4 and 5-6 different antibiotics. Diagonal lines represent a significant difference compared to the control (NAB) on the same day. (A) Cloacal swabs on day 31 (NAB *n* = 47, BMD *n* = 47, EFX *n* = 31, BMD_EFX *n* = 72). (B) Cloacal swabs on day 41 (NAB *n* = 48, BMD *n* = 37, EFX *n* = 48, BMD_EFX *n* = 40). (C) Litter on day 31 (NAB *n* = 30, BMD *n* = 29, EFX *n* = 31, BMD_EFX *n* = 40). (D) Litter on day 41 (NAB *n* = 28, BMD *n* = 32, EFX *n* = 25, BMD_EFX *n* = 20). **Figure S19.** Relative abundance (%) of multidrug resistant ESBL-E isolates from day 27, resistant to 0, 3-4, 5-6 and 7-11 different antibiotics. (A) Cloacal swabs, NAB (*n* = 113), BMD (*n* = 96). (B) Litter, NAB (*n* = 155), BMD (*n* = 113). **Figure S20.** Relative abundance (%) of multidrug resistant ESBL-E isolates from days 31 and 41 resistant to 0, 3-4, 5-6 and 7-11 different antibiotics. Diagonal lines represent a significant difference compared to the control (NAB) on the same day. (A) Cloacal swabs on day 31 (NAB *n *= 56, BMD *n* = 48, EFX *n* = 2, BMD_EFX *n* = 32). (B) Cloacal swabs on day 41 (NAB *n* = 48, BMD *n* = 40, EFX *n* = 34, BMD_EFX *n* = 33). (C) Litter on day 31 (NAB *n* = 36, BMD *n* = 34, EFX *n* = 45, BMD_EFX *n* = 29). (D) Litter on day 41 (NAB *n *= 21, BMD *n* = 24, EFX *n* = 10, BMD_EFX *n* = 20).

**Additional file 2: Table S1.** Overview of metagenomic sequencing data. **Table S2.** Metagenome Assembled Genomes (MAGs) recovered from cloacal swab and litter samples. **Table S3.** Representative high quality MAGs recovered from cloacal swab (*n* = 26) and litter (*n* = 12) samples. **Table S4.** Representative MAGs associated with pathogen-harboring families from cloacal swab (*n* = 17) and litter (*n* = 7) samples. **Table S5.** Presence (1) of ARG subtypes in cloacal swab and litter samples. **Table S6.** Presence (1) of specific ARG subtypes in MAGs from pathogen-harboring families (17 Cloacal swab and 7 litter MAGs). **Table S7.** List of ARGs and MGEs targeted in High throughput qPCR array analysis.

## Data Availability

The sequencing data generated from both shotgun metagenomes and amplicon sequencing for this study has been deposited to the NCBI SRA database under the primary accession number Bioproject ID: PRJNA598014. Additional data can be shared upon request.

## Abbreviations

AMR: Antimicrobial resistance
AGP: Antibiotic growth promoter
NAB: No antibiotic
BMD: Bacitracin
EFX: Enrofloaxacin
ARB: Antibiotic-resistant bacteria
ARG: Antibiotic resistance gene
MGE: Mobile genetic element
MDR: Multidrug resistance
MRSA: Methicillin-resistant *Staphylococcus aureus*
VRE: Vancomycin-resistant *Enterococcus*
ESBL-E: Extended-spectrum beta-lactamase-producing *Enterobacteriaceae*
NMDS: Non-metric multidimensional scaling
MLS: Macrolide-lincosamide-streptogramin
MAGs: Metagenome-assembled genomes
