# Supplementary material for: Longitudinal study on the effects of growth-promoting and therapeutic antibiotics on the dynamics of chicken cloacal and litter microbiomes and resistomes
Source: Microbiome. 2021 Aug 28;9:178. doi: 10.1186/s40168-021-01136-4 (PMC8403378; doi:10.1186/s40168-021-01136-4)
Supplement: Supplementary file 2 — Additional file 1: Figure S1. Temporal increase in body weight of broiler chickens for the duration of the growth cycle. Left side, chickens fed with or without BMD (NAB) up to day 27. Right side, body weight immediately after enrofloxacin treatment (EFX and BMD_EFX) on day 41. Figure S2. Individual production parameters: body weight on day of slaughter and meat processing parameters (relative breast, heart, liver and abdominal fat weights) after slaughter of chicken in the four different treatment types. a-b indicate statistical significant differences (p < 0.05). Figure S3. Distribution of microbial domains in cloacal swab and litter (A); and the relative abundance of prominent non-bacterial (Archaea, Eukaryota and Viruses) families (B) as a function of sampling time and treatment. The abundance data was normalized by scaling each row separately to emphasize abundance as a function of treatment. Figure S4. Diversity and bacterial community composition in litter derived from 16S rRNA gene amplicon sequencing. (A) Temporal fluctuations in alpha diversity (Shannon index). Values are presented as the median (black horizontal line), lower and upper hinges correspond to the 25th and 75th percentiles and the outliers are displayed by small black dots; and (B) relative abundance of bacterial families; unclassified taxa and bacterial families having relative abundance less than 0.5% were grouped into “Others”. The relative abundance of each family was represented as the mean value of the biological triplicates of corresponding samples. *p < 0.05, and n.s. indicates p > 0.05 by Kruskal-Wallis test followed by pairwise comparison using Dunn’s post-hoc method. Figure S5. Temporal changes in the distribution of the three-targeted pathogenic-associated bacterial families: (A) Enterobacteriaceae, (B) Entercococaceae, and (C) Staphylococaceae, in litter derived from 16S rRNA gene amplicon sequencing data. The relative abundance values are presented as the median (black horizontal [file 40168_2021_1136_MOESM1_ESM.docx]

**Longitudinal study on the effects of growth promoting and therapeutic antibiotics on the dynamics of chicken cloacal and litter microbiomes and resistomes**

**Gupta et al.**

**Supplementary Figures**


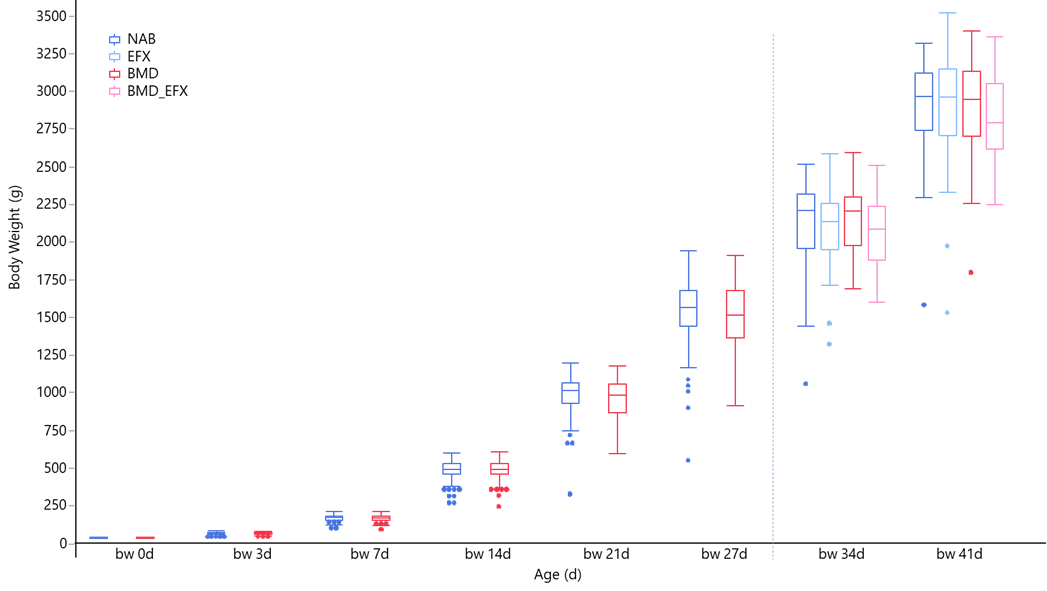


***Figure S1****: Temporal increase in body weight of broiler chickens for the duration of the growth cycle. Left side, chickens fed with or without BMD (NAB) up to day 27. Right side, body weight immediately after enrofloxacin treatment (EFX and BMD_EFX) on day 41.*


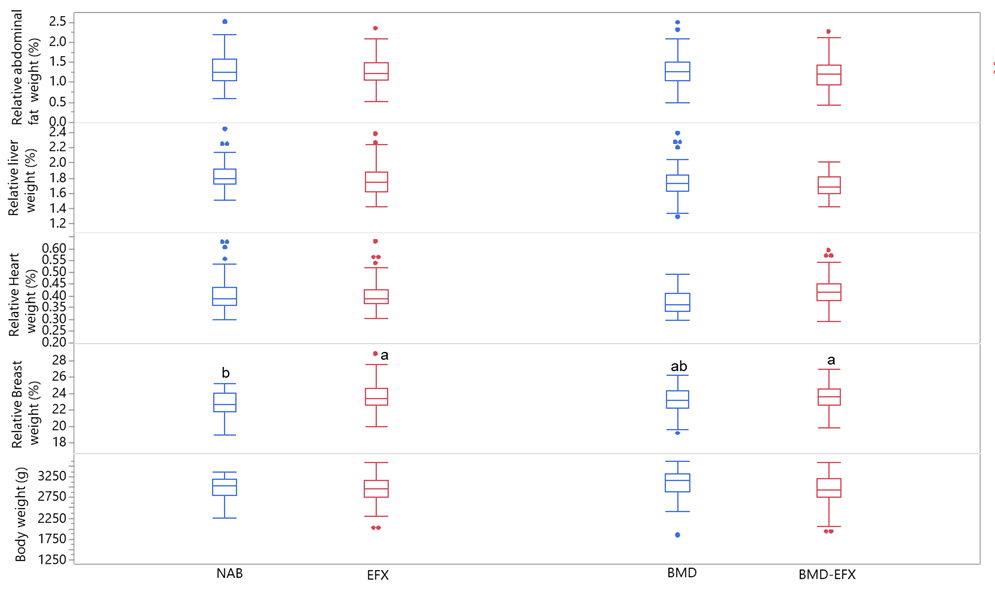


***Figure S2****: Individual production parameters: body weight on day of slaughter and meat processing parameters (relative breast, heart, liver and abdominal fat weights) after slaughter of chicken in the four different treatment types. a-b indicate statistical significant differences (p<0.05).*

*
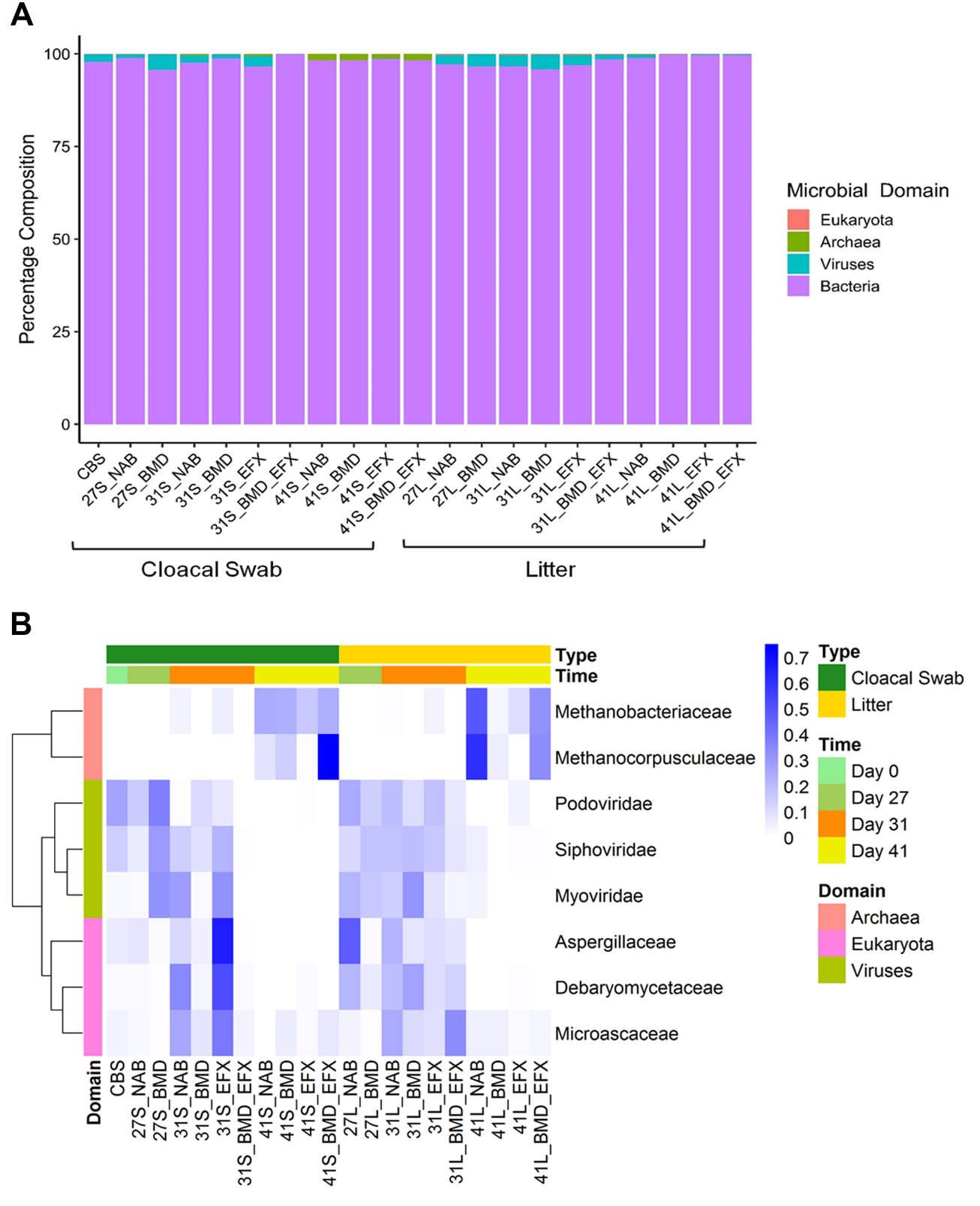
*

***Figure S3:*** *Distribution of microbial domains in cloacal swab and litter (A); and the relative abundance of prominent non-bacterial (Archaea, Eukaryota and Viruses) families (B) as a function of sampling time and treatment. The abundance data was normalized by scaling each row separately to emphasize abundance as a function of treatment.*


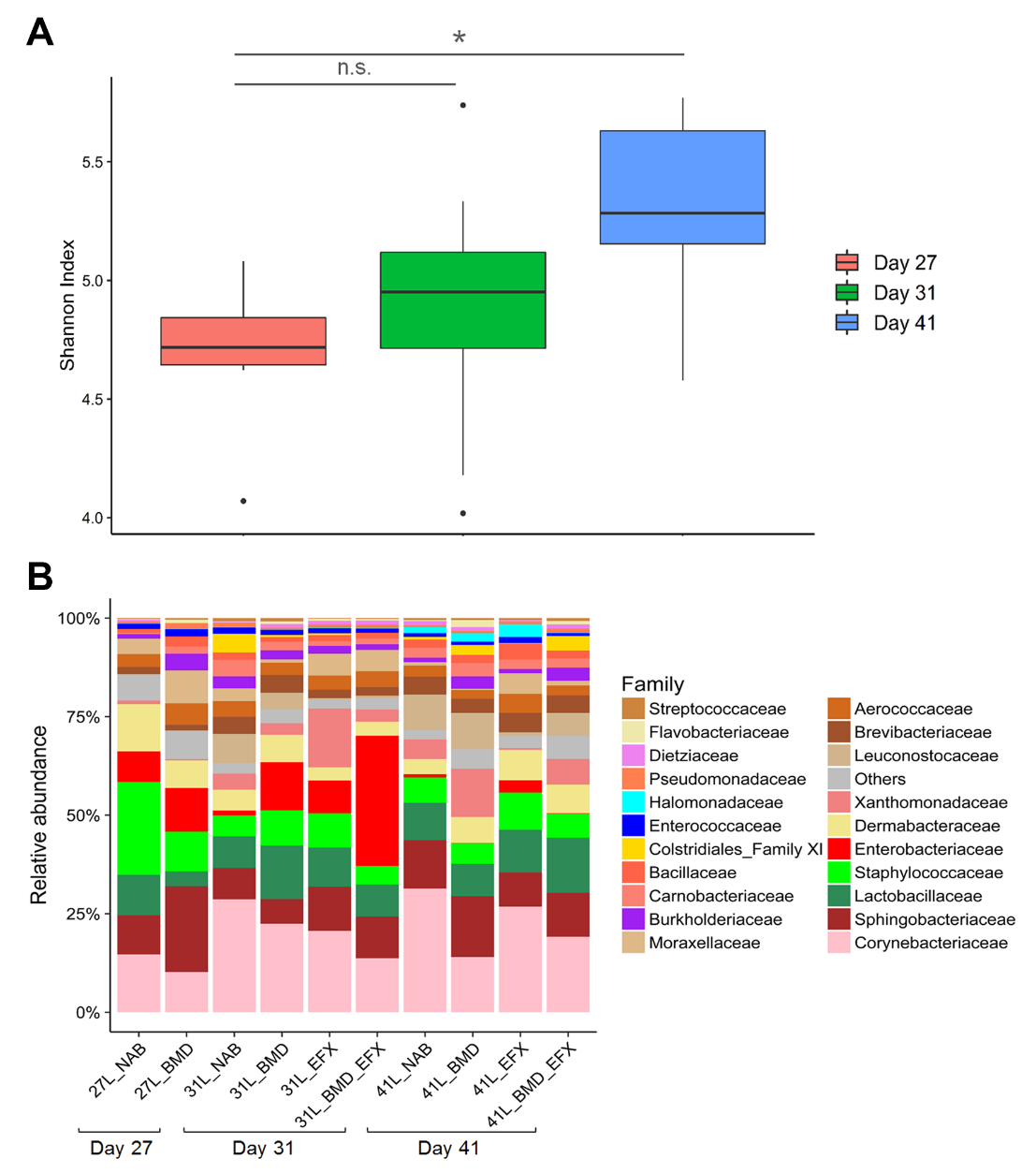


***Figure S4:*** *Diversity and bacterial community composition in litter derived from 16S rRNA gene amplicon sequencing. (A) Temporal fluctuations in alpha diversity (Shannon index). Values are presented as the median (black horizontal line), lower and upper hinges correspond to the 25^th^ and 75^th^ percentiles and the outliers are displayed by small black dots; and (B) relative abundance of bacterial families; unclassified taxa and bacterial families having relative abundance less than 0.5% were grouped into “Others”. The relative abundance of each family was represented as the mean value of the biological triplicates of corresponding samples. *p<0.05, and n.s. indicates p>0.05 by Kruskal-Wallis test followed by pairwise comparison using Dunn’s post-hoc method.*

*
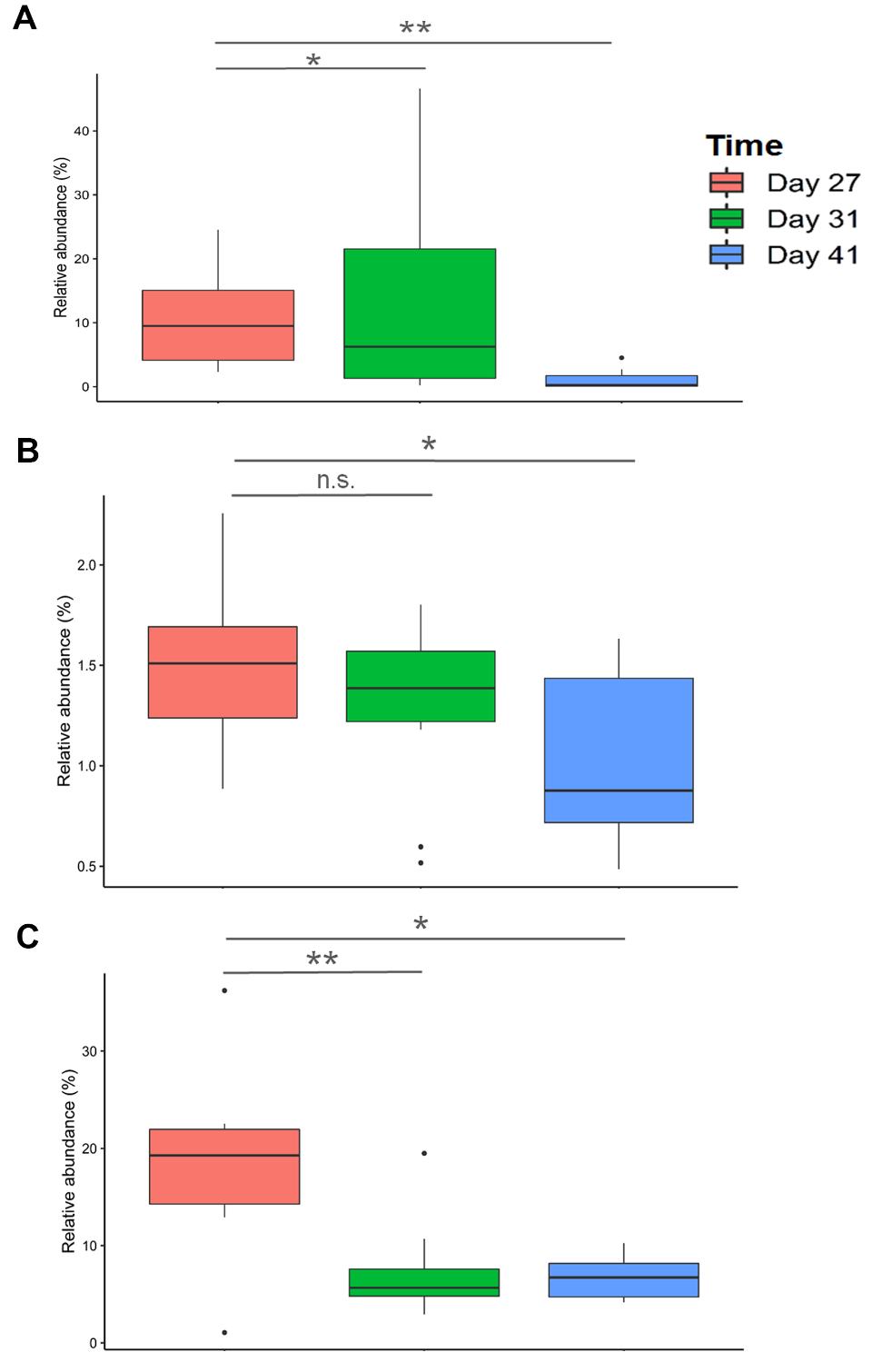
*

***Figure S5:*** *Temporal changes in the distribution of the three-targeted pathogenic-associated bacterial families: (A) Enterobacteriaceae, (B) Entercococaceae, and (C) Staphylococaceae, in litter derived from 16S rRNA gene amplicon sequencing data. The relative abundance values are presented as the median (black horizontal line), lower and upper hinges corresponds to the 25^th^ and 75^th^ percentiles. The outliers are displayed by small black dots. *p <0.05, **p <0.01, and n.s. indicates p>0.05, by Friedman’s test followed by pairwise comparison using Nemenyi pot-hoc test.*


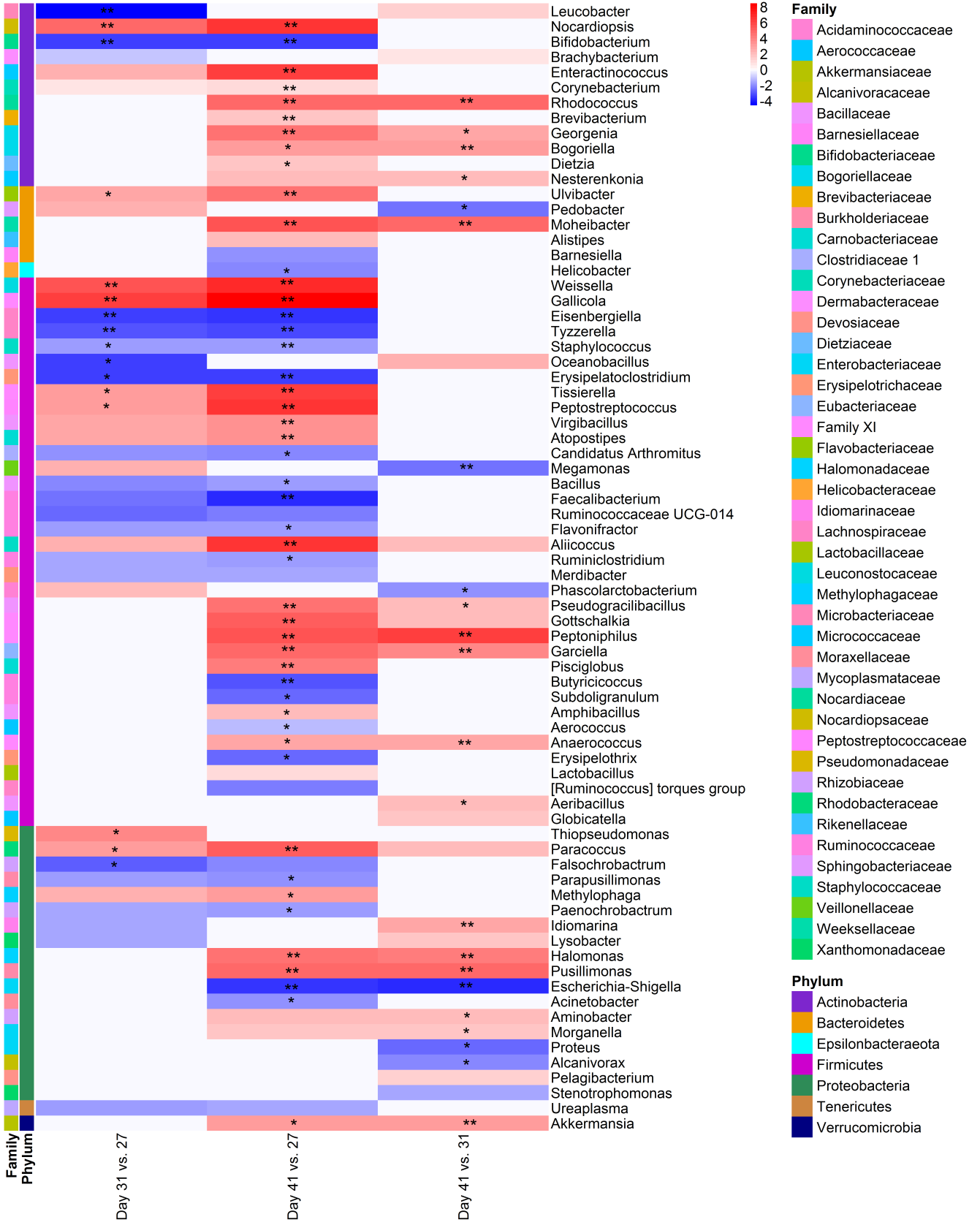


***Figure S6A****: Differentially abundant bacterial genera (derived from 16S rRNA gene amplicon sequencing) observed in chicken litter over the time. The Log2Fold change value of bacterial genus at padj (adjusted p-value) <0.10 are presented here and significant Log2Fold change with padj <0.05 and <0.01 were marked by single and double stars respectively. The red and blue boxes represents enriched and suppressed genera over the time. Bacterial genus are also grouped by their family and phylum.*

*
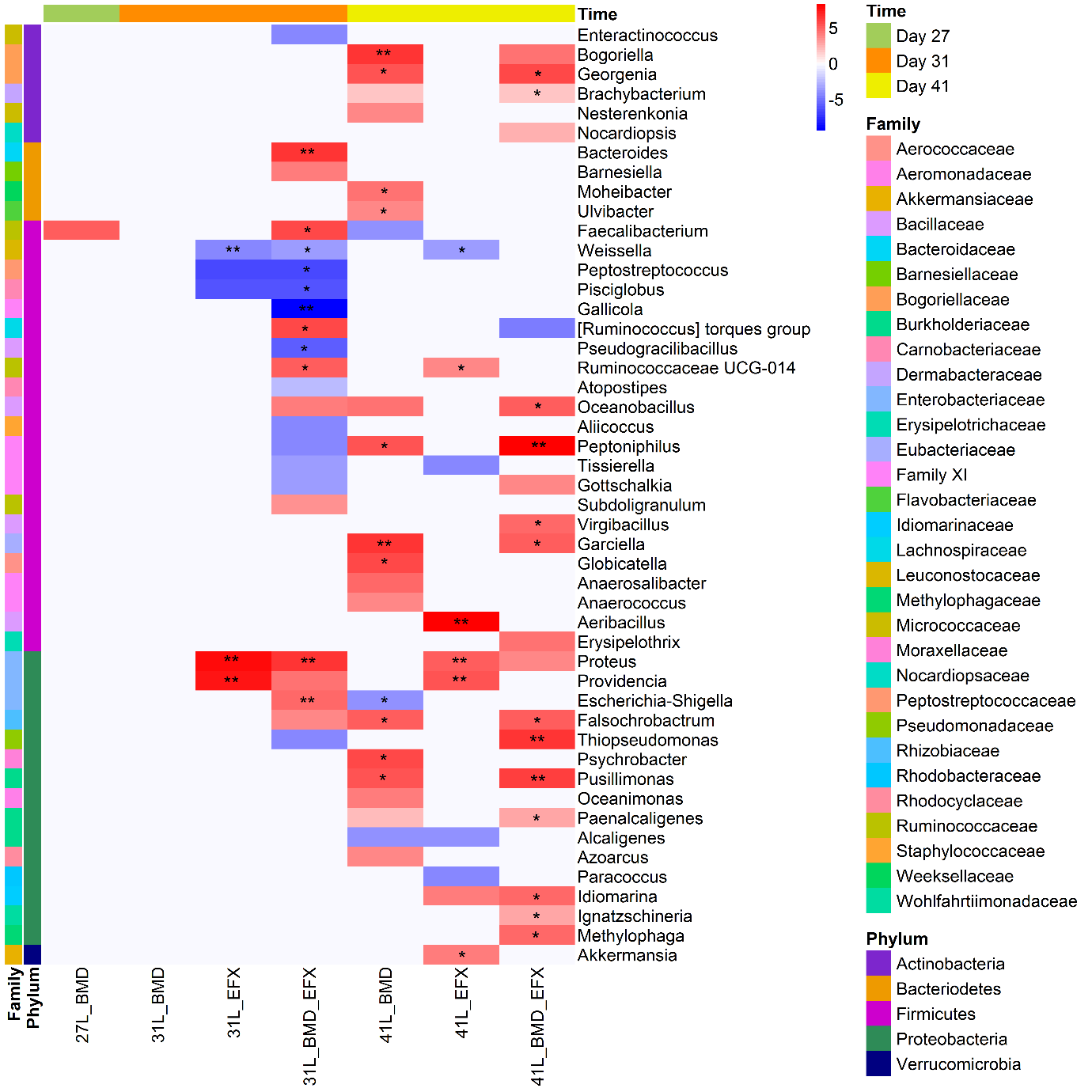
*

***Figure S6B:*** *Litter-associated bacterial genera positively or negatively correlated to antibiotic treatments derived from 16S rRNA gene amplicon sequencing. Significant Log2Fold changes (antibiotic treatment relative to corresponding NAB samples) with padj <0.05 and <0.01 are marked by single and double stars respectively. Red and blue boxes represent antibiotic-stimulated and antibiotic-suppressed genera, respectively.*

*
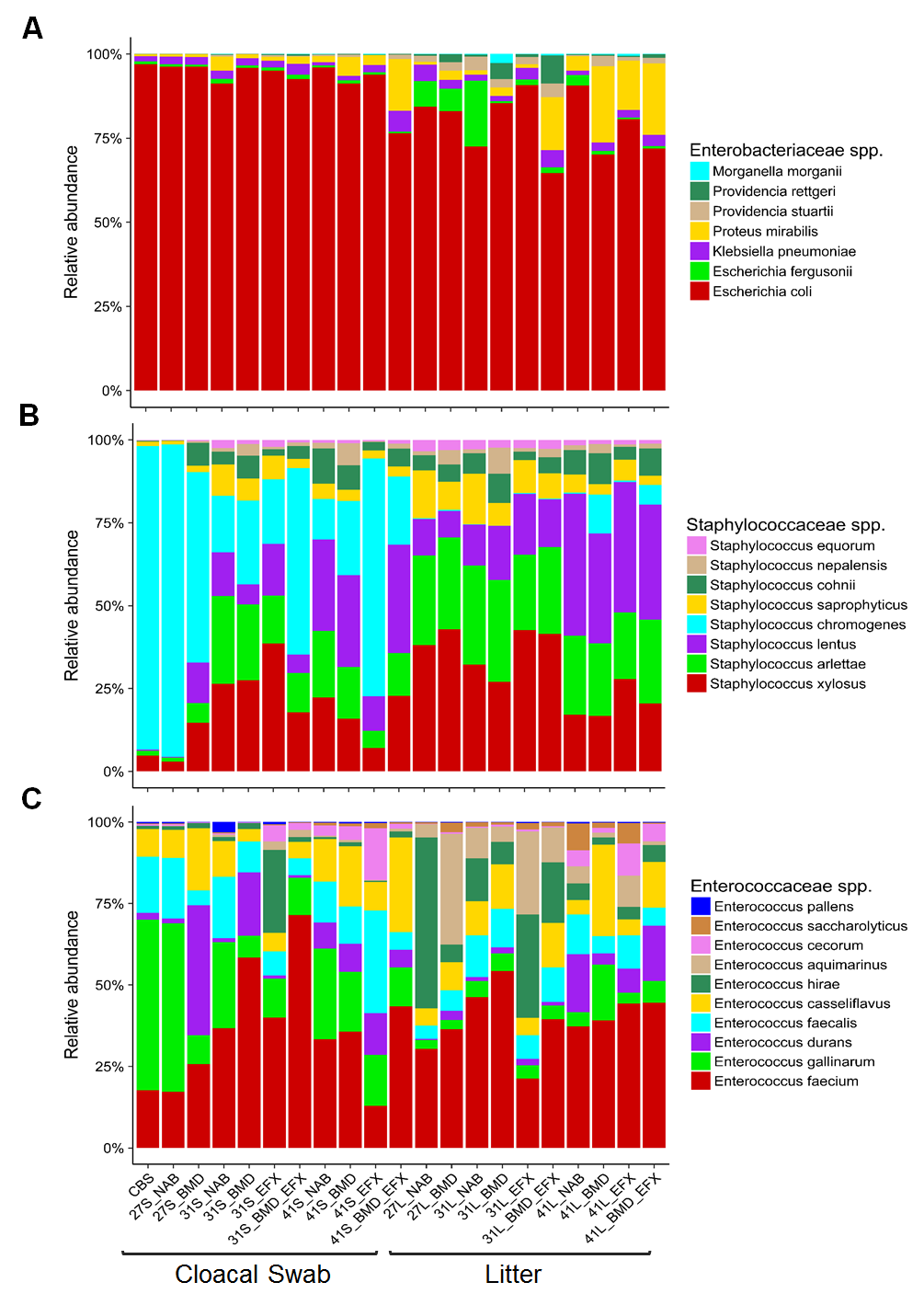
*

***Figure S7:*** *Relative distribution of bacterial species within (A) Enterobacteriaceae, (B) Staphylococcaceae, and (C) Enterococcaceae families in the cloacal swab and litter metagenomes.*

*
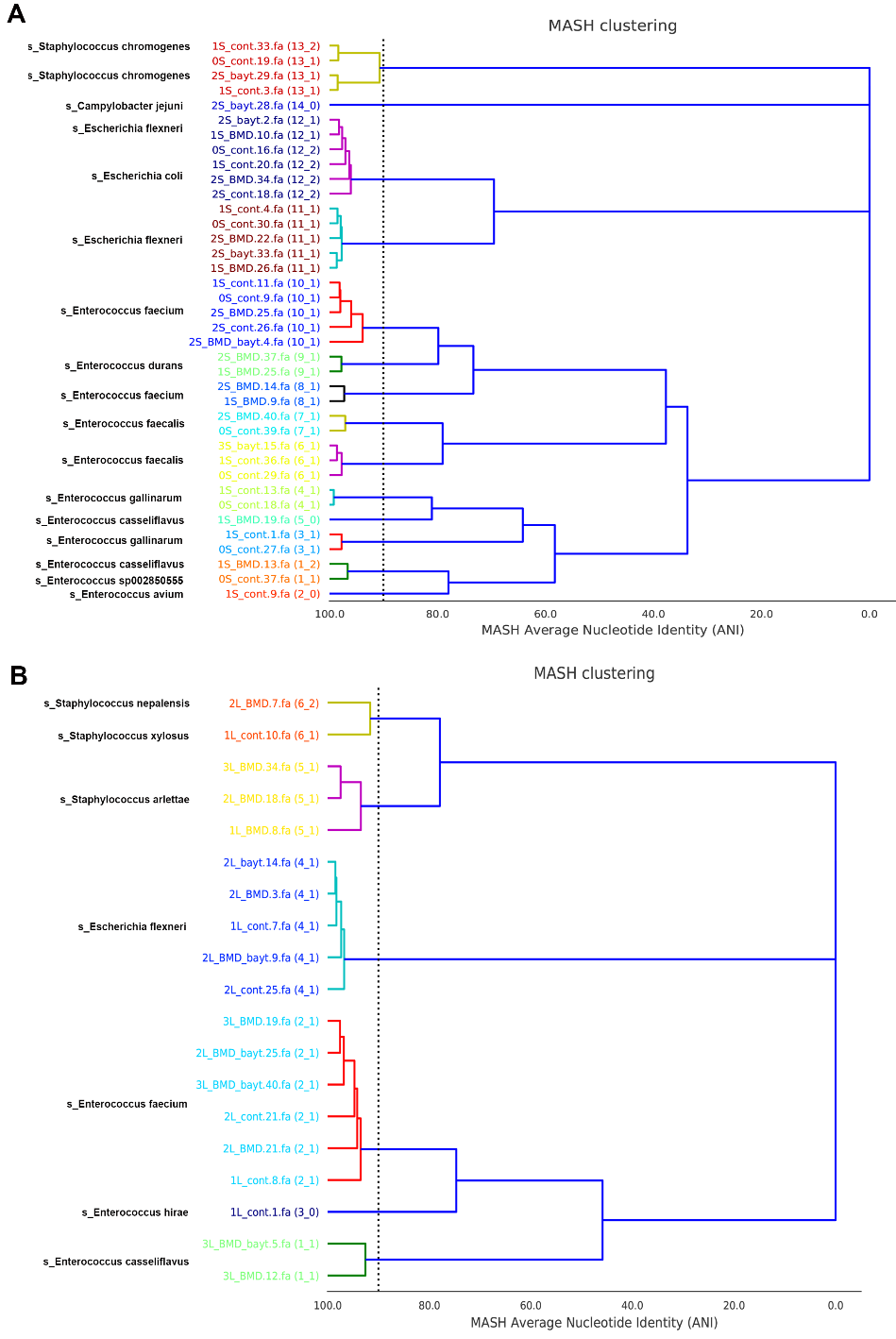
*

***Figure S8:*** *Dendrogram generated from MASH analysis (dRep workflow) of potential pathogen-associated family MAGs recovered from (A) cloacal swabs (n=38) and (B) litter (n=19). The de-replication analysis suggested a reduced set of 17 and 7 representative MAGs for swab and litter, respectively.*

*
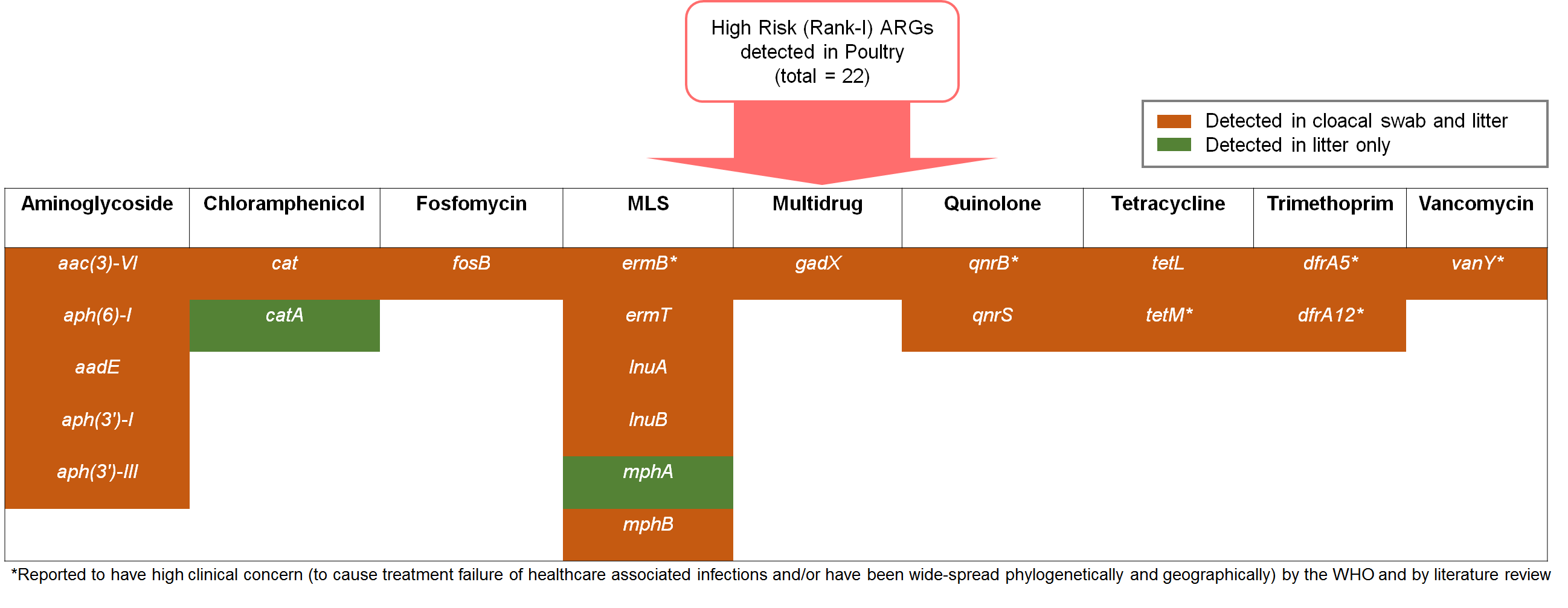
*

***Figure S9:*** *Priority (Rank-I) ARGs in cloacal swab and litter samples based on ARG-ranker (*[*https://github.com/caozhichongchong/arg_ranker*](https://github.com/caozhichongchong/arg_ranker)*) predictions.*

*
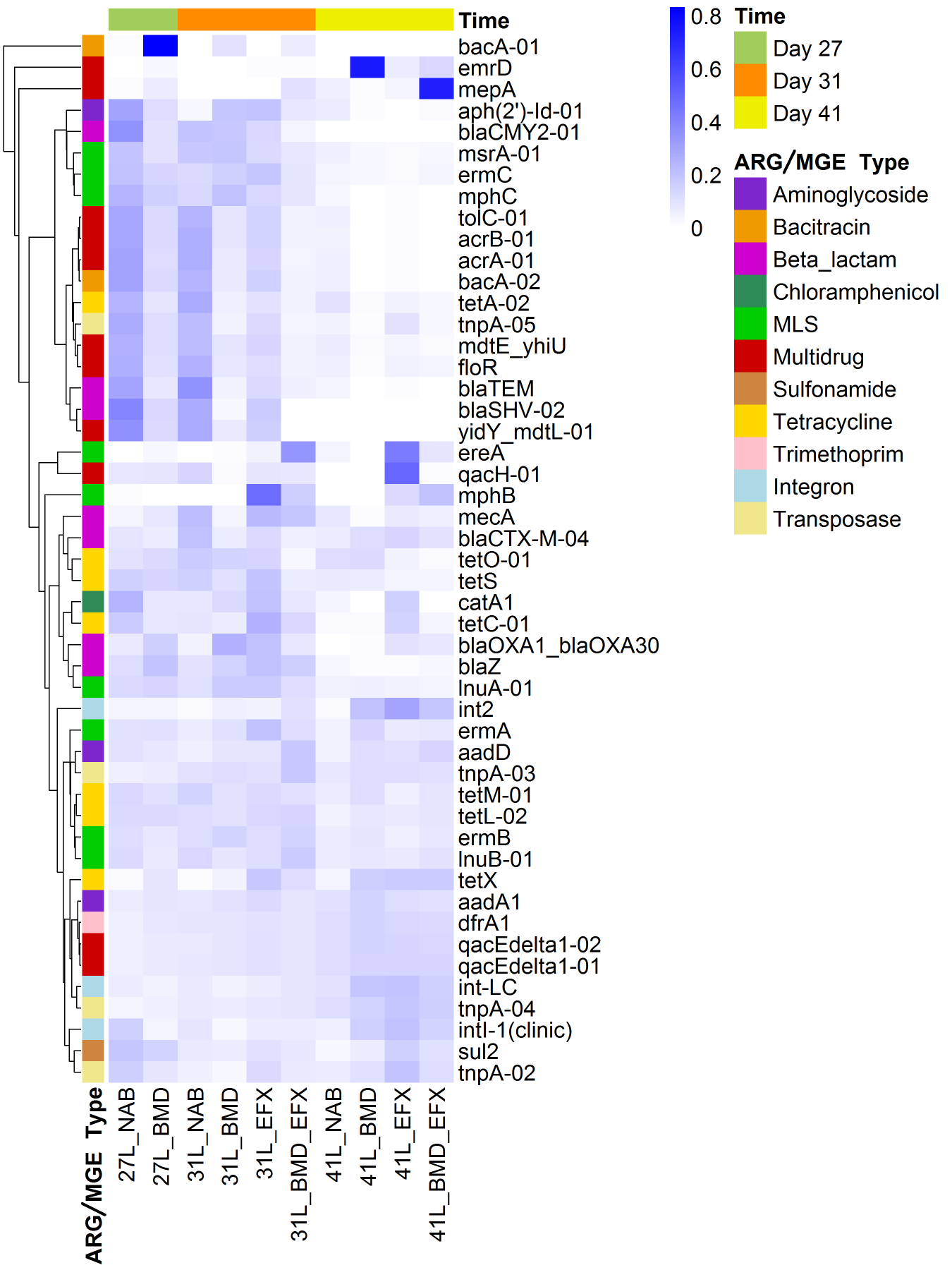
*

***Figure S10:*** *Relative abundance of ARGs and MGEs in chicken litter samples obtained from HTqPCR (High-throughput quantitate PCR) data analysis. The mean abundance of triplicate biological samples was normalized by scaling each row separately to better visualize the impact of individual treatments.*

*
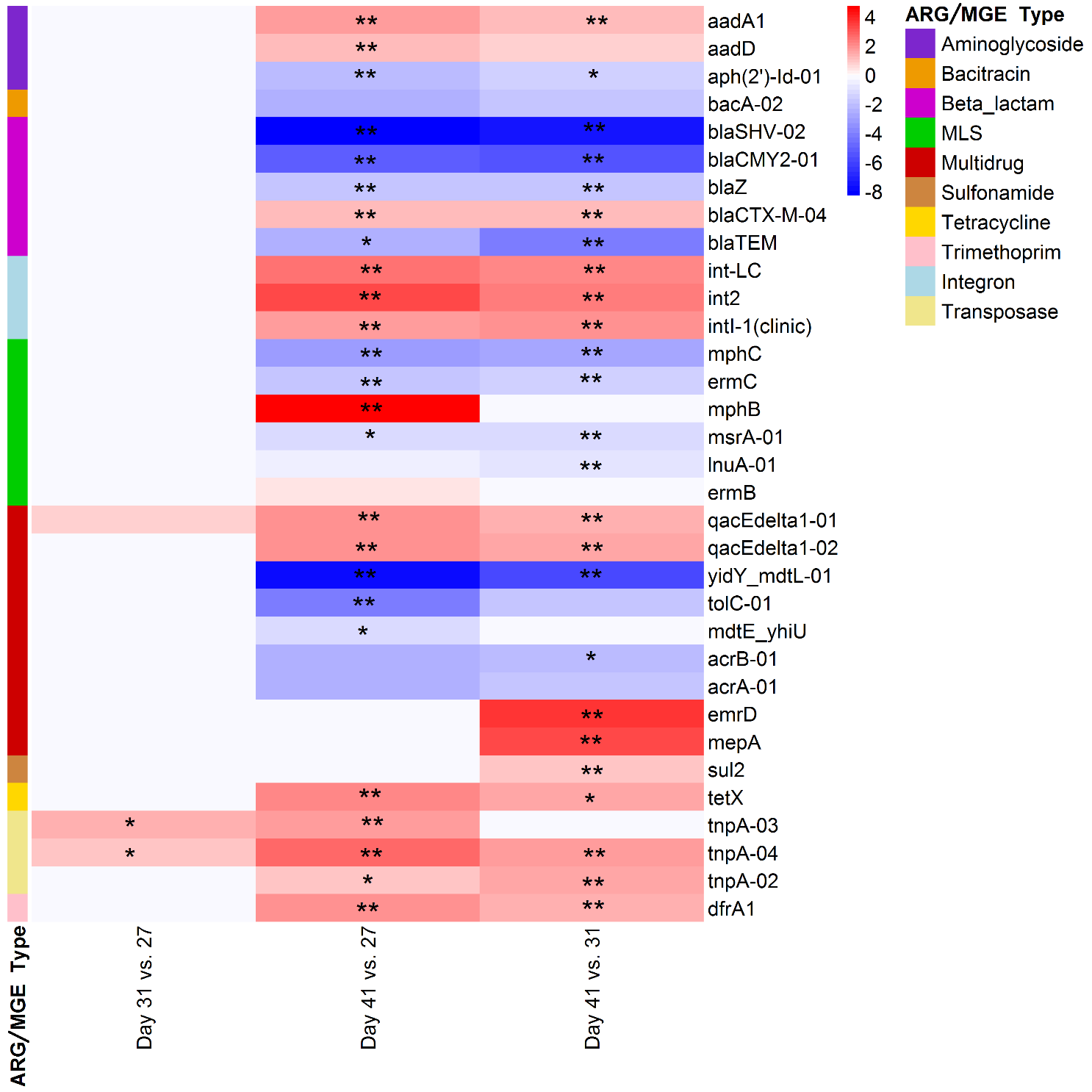
*

***Figure S11A:*** *Differentially abundant ARGs and MGEs (derived from HT-qPCR analysis) observed in chicken litter over time. The Log2Fold change of ARG/MGE values with padj (adjusted p-value) <0.10 are presented, and significant Log2Fold change with padj <0.05 and <0.01 are marked by single and double stars respectively. The red and blue boxes represent ARGs/MGEs that significantly increase and decrease over the time. ARGs/MGEs are also grouped by category.*

**
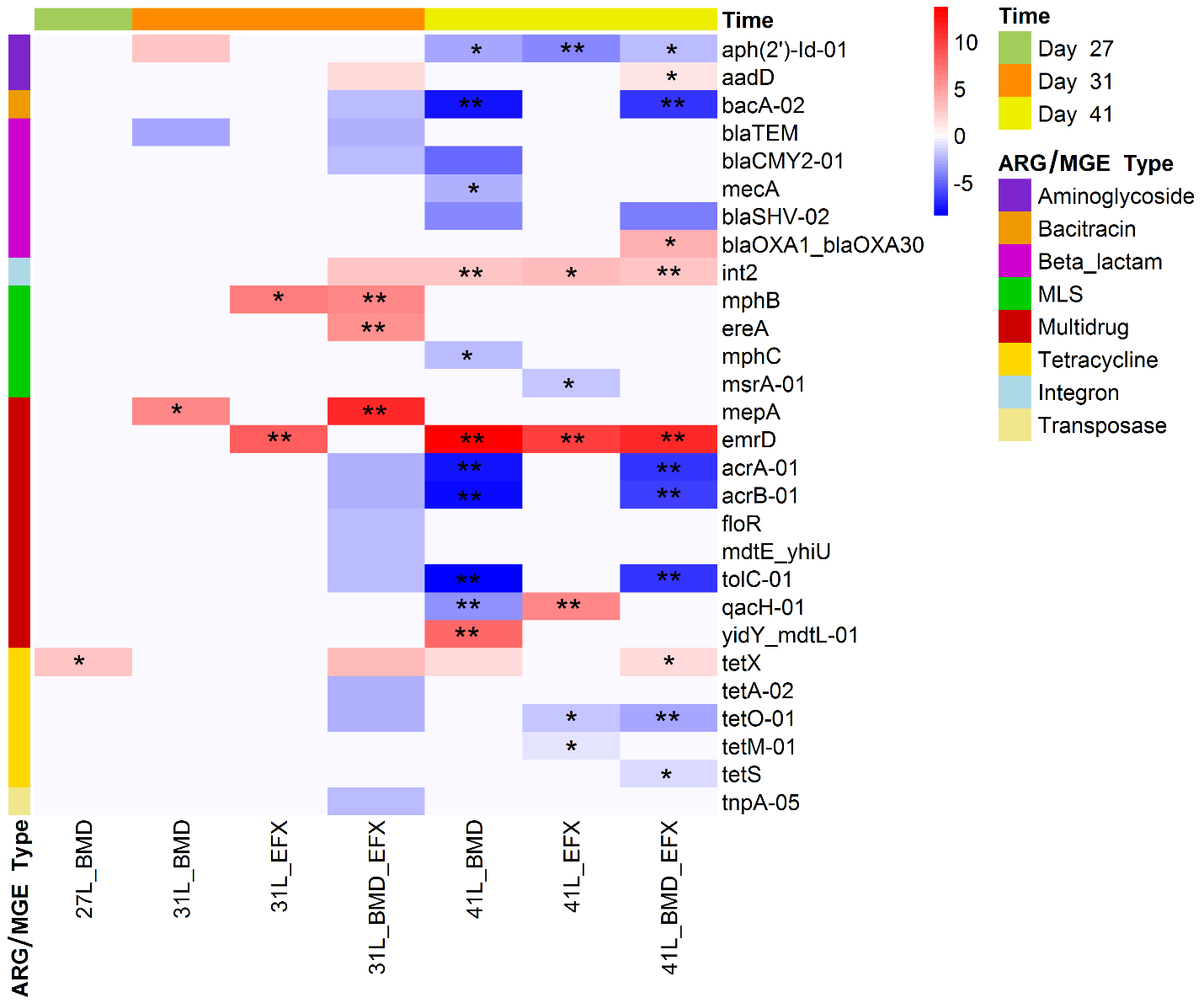
**

***Figure S11B:*** *Litter-associated ARGs and MGEs positively or negatively correlated to antibiotic treatments derived from HT-qPCR analysis. Significant Log2Fold changes (antibiotic treatment relative to corresponding NAB samples) with. padj <0.05 and <0.01 are marked by single and double stars respectively. Red and blue boxes represent antibiotic-stimulated and antibiotic-suppressed ARGs/MGEs, respectively.*

*
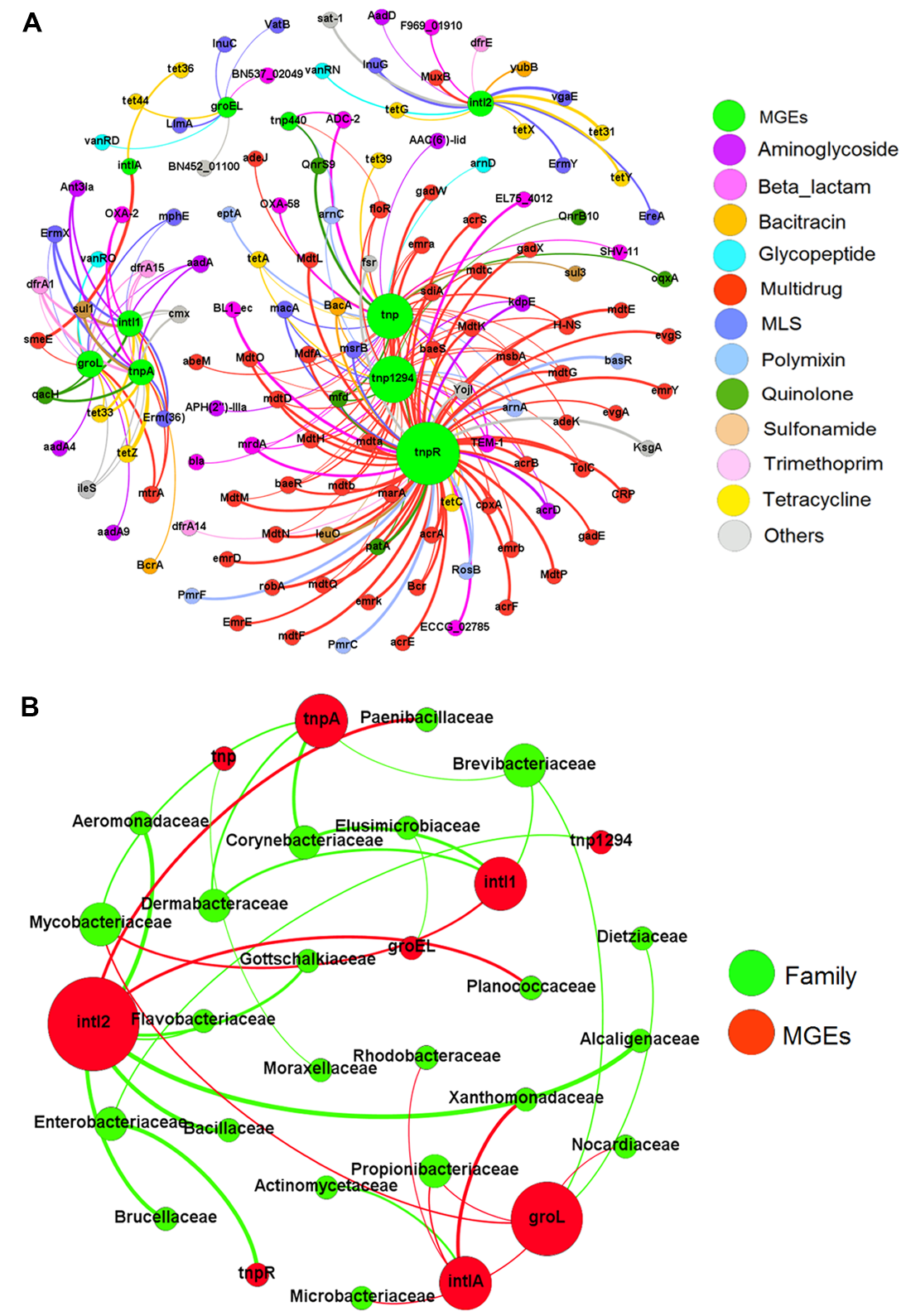
*

***Figure S12:*** *Network analysis showing correlations between (A) ARGs and MGEs, and (B) MGEs and bacterial families. Nodes show ARGs, MGEs and bacterial families and edges (i.e., connections between ARG and MGE, or MGE and family) indicate significant (padj <0.01) and strong pairwise correlations (spearman rho >0.80). The size of each node is proportional to the number of connections (i.e. degree) and the edge thickness is proportional to the spearman correlation coefficient (rho; 0.80-0.99). MLS: Macrolide-Lincosamide-Streptogramin; MGEs: mobile genetic elements.*

*
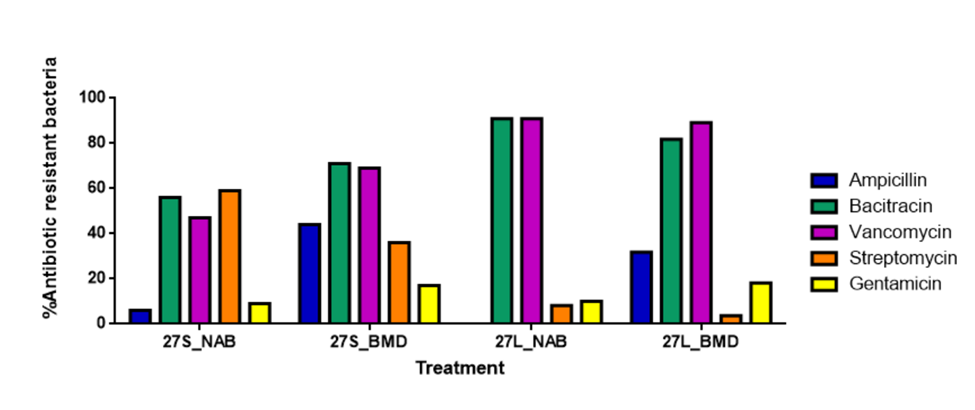
*

***Figure S13:*** *Relative abundance (%) of VRE isolates from day 27 resistant to additional antibiotics. Cloacal swabs: 27S_NAB (n=48), 27S_BMD (n=78). Litter: 27L_NAB (n=34), 27L_BMD (n=48).*

*
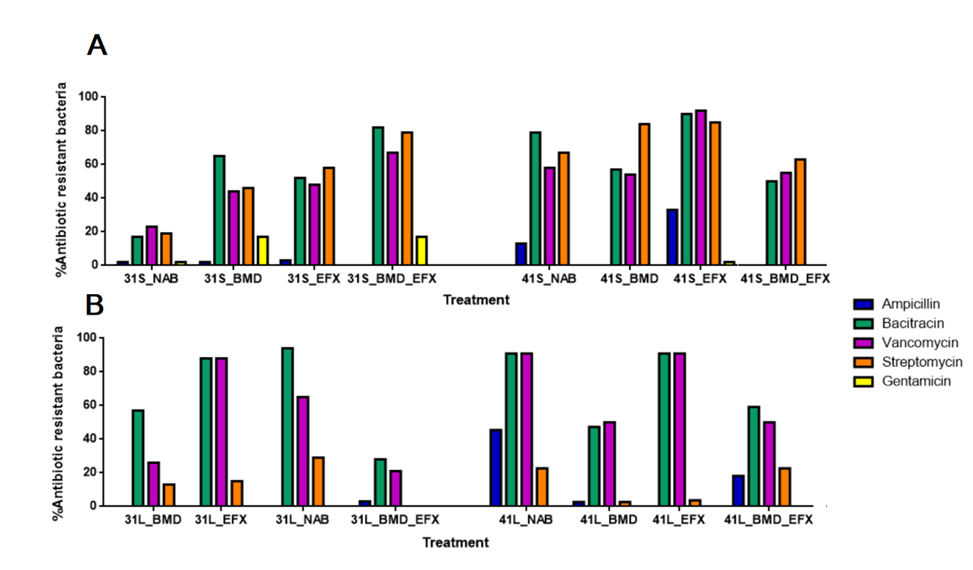
*

***Figure S14:*** *Relative abundance (%) of VRE isolates resistant to additional antibiotics from (A) cloacal swabs and (B) litter. Cloacal swabs: NAB (31d n=48, 41d n=47), BMD (31d n=37, 41d n=48), EFX (31d n=48, 41d n=31), BMD_EFX (31d n=40, 41d n=72). Litter: NAB (31d n=17, 41d n=28), BMD (31d n=23, 41d n=31), EFX (31d n=36, 41d n=24), BMD_EFX (31d n=29, 41d n=20).*

*
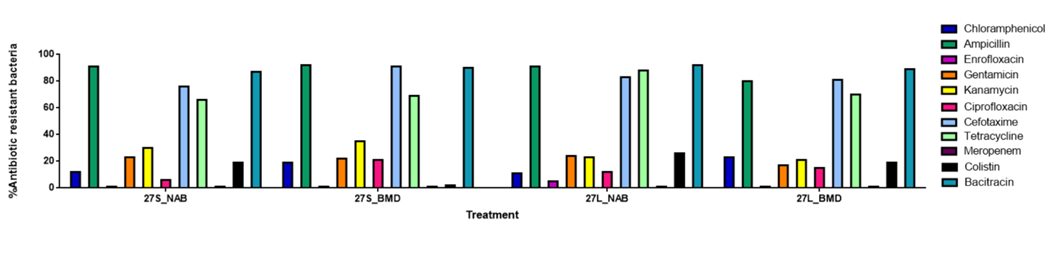
*

***Figure S15:*** *Relative abundance (%) of ESBL-E isolates resistant to additional antibiotics isolated on day 27. Cloacal swabs: 27S_NAB (n=113), 27S_BMD (n=96). Litter: 27S_NAB (n=155), 27S_BMD (n=113).*

*
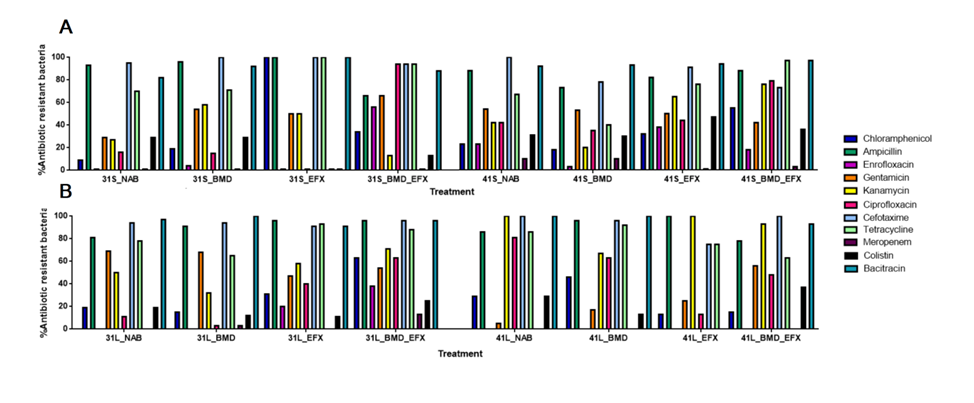
*

***Figure S16:*** *Relative abundance (%) of ESBL-E isolates resistant to additional antibiotics from (A) cloacal swabs and (B) litter. Cloacal swabs: NAB (31d n=56, 41d n=48), BMD (31d n=48, 41d n=40), EFX (31d n=2, 41d n=34), BMD_EFX (31d n=32, 41d n=33). Litter: NAB (31d n=36, 41d n=21), BMD (31d n=34, 41d n=24), EFX (31d n=45, 41d n=10), BMD_EFX (31d n=29, 41d n=20).*

*
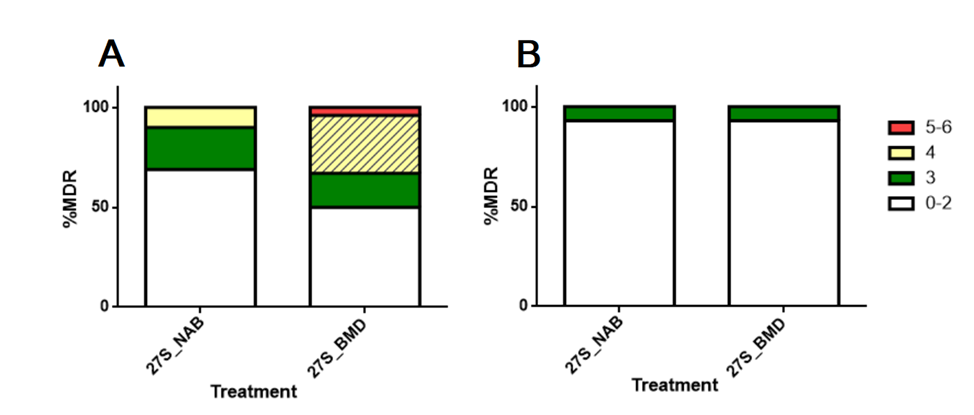
*

***Figure S17:*** *Relative abundance (%) of multidrug resistant VRE isolates isolated on day 27, resistant to 0-2, 3, 4 and 5-6 different antibiotics. Diagonal lines represent a significant difference compared to the control (NAB) on the same day. Cloacal swabs: 27S_NAB (n=48), 27S_BMD (n=78). Litter: 27L_NAB (n=34), 27L_BMD (n=48).*

*
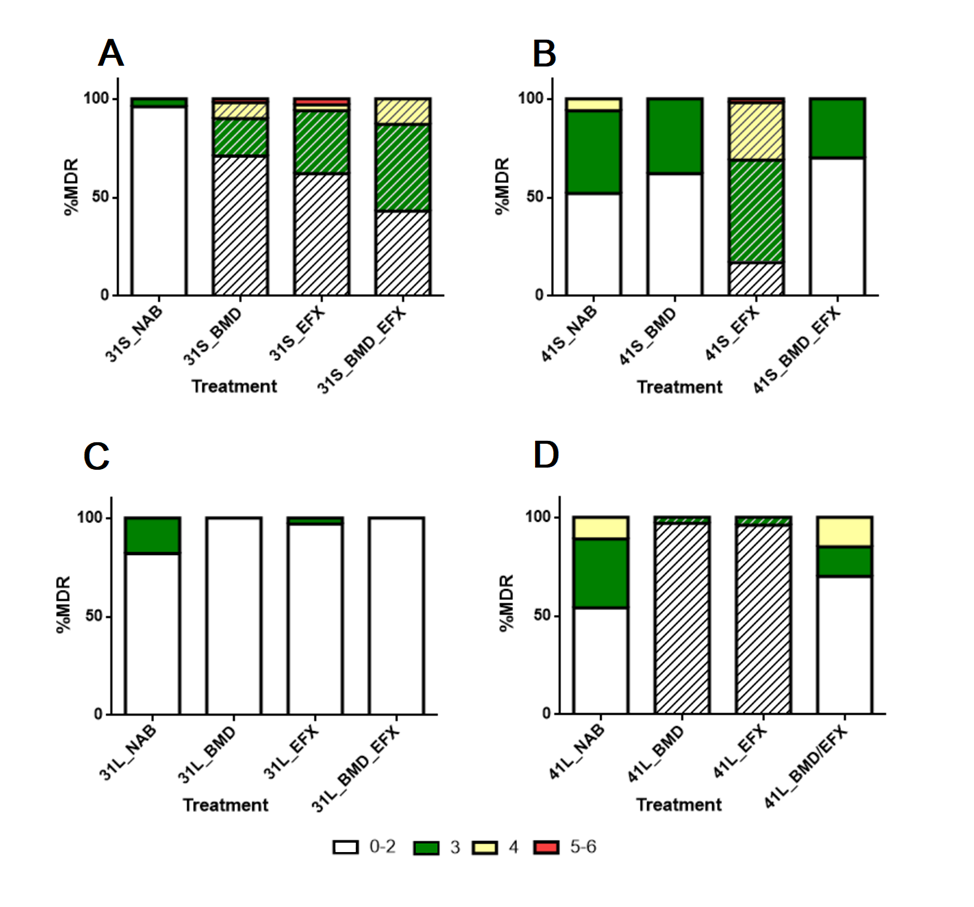
*

***Figure S18:*** *Relative abundance (%) of multidrug resistant VRE isolates from days 31 and 41, resistant to 0-2, 3, 4 and 5-6 different antibiotics. Diagonal lines represent a significant difference compared to the control (NAB) on the same day. (A) Cloacal swabs on day 31 (NAB n=47, BMD n=47, EFX n=31, BMD_EFX n=72). (B) Cloacal swabs on day 41 (NAB n=48, BMD n=37, EFX n=48, BMD_EFX n=40). (C) Litter on day 31 (NAB n=30, BMD n=29, EFX n=31, BMD_EFX n=40). (D) Litter on day 41 (NAB n=28, BMD n=32, EFX n=25, BMD_EFX n=20).*

*
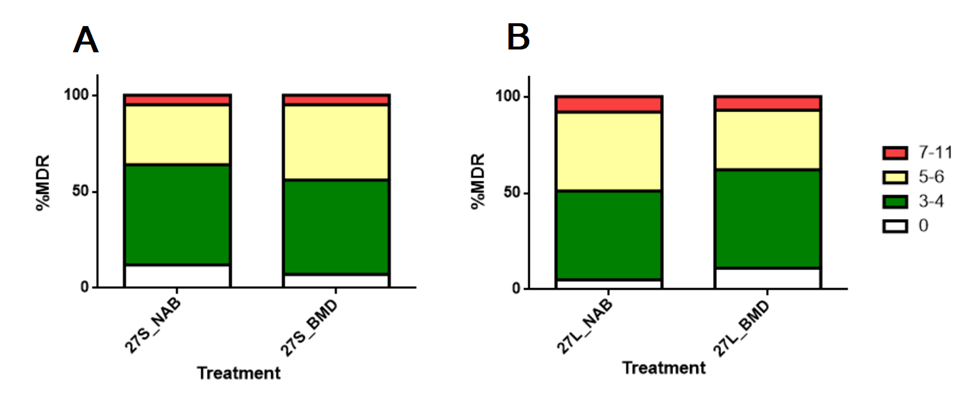
*

***Figure S19:*** *Relative abundance (%) of multidrug resistant ESBL-E isolates from day 27, resistant to 0, 3-4, 5-6 and 7-11 different antibiotics. (A) Cloacal swabs, NAB (n=113), BMD (n=96). (B) Litter, NAB (n=155), BMD (n=113).*


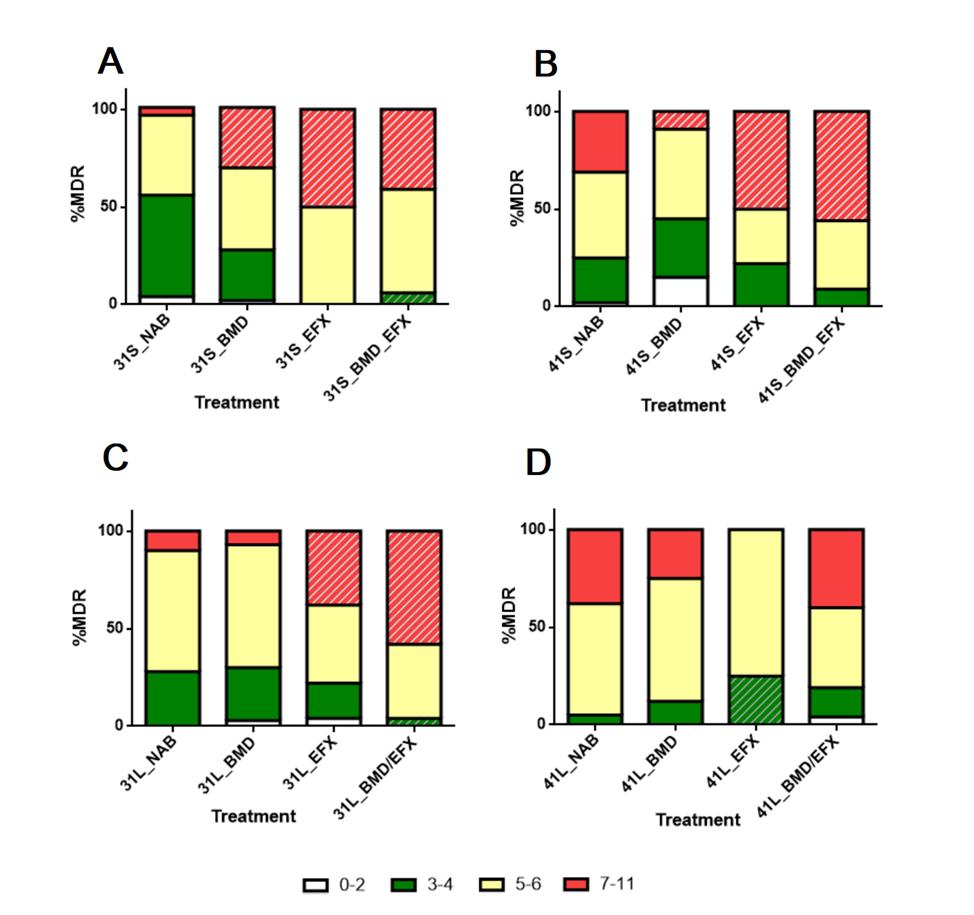


***Figure S20:*** *Relative abundance (%) of multidrug resistant ESBL-E isolates from days 31 and 41 resistant to 0, 3-4, 5-6 and 7-11 different antibiotics. Diagonal lines represent a significant difference compared to the control (NAB) on the same day. (A) Cloacal swabs on day 31 (NAB n=56, BMD n=48, EFX n=2, BMD_EFX n=32). (B) Cloacal swabs on day 41 (NAB n=48, BMD n=40, EFX n=34, BMD_EFX n=33). (C) Litter on day 31 (NAB n=36, BMD n=34, EFX n=45, BMD_EFX n=29). (D) Litter on day 41 (NAB n=21, BMD n=24, EFX n=10, BMD_EFX n=20).*
